# High-precision crop recommendation system with stacking ensemble classifiers for optimizing agricultural productivity

**DOI:** 10.1038/s41598-025-09640-5

**Published:** 2025-12-09

**Authors:** Rania A. Ahmed, Walid El-Shafai, Zeinab A. Ahmed, El-Sayed M. El-Rabaie, Fathi E. Abd El-Samie

**Affiliations:** 1 Climate Change Information Center and Renewable Energy and Expert System, Agricultural Research Center (ARC), Giza, Egypt; 2 Department of Electronics and Electrical Communications Engineering, Faculty of Electronic Engineering, Menoufia University, Menouf, 32952 Egypt; 3 Automated Systems and Computing Lab (ASCL), Department of Computer Science, Prince Sultan University, 11586 Riyadh, Saudi Arabia; 4 Department of Information Technology, College of Computer and Information Sciences, Princess Nourah bint Abdulrahman University, P.O. Box 84428, 11671 Riyadh, Saudi Arabia

**Keywords:** Ensemble classifier, Crop recommendation system, ML, Bagging, Boosting, Stacking, Plant sciences, Mathematics and computing

## Abstract

Crop productivity is crucial for farmers and economy worldwide. Factors such as fertilization, weather, and climate have a significant impact on yields. To improve crop productivity, a crop recommendation system is introduced in this paper. It provides data-driven advice on the best crops to plant, taking into account climate, weather, and soil nutrients. This research work introduces feature fusion with a stacking ensemble model comprising 18 classifiers and three novel methods to enhance crop recommendation and mitigate overfitting compared to other ensemble techniques. In this paper, we also examine two datasets for model validation; one of them is a large dataset containing nearly 28,242 records. The findings of our study reveal that feature fusion enables all ensemble classifiers to not only exceed the accuracy and precision of other established modern techniques, but also reduce overfitting, especially for the three proposed models that depend on a large dataset. In our experiments, the accuracy of ensemble models in categorizing diverse crops under different conditions ranges from 98.4% to 99.54%. Notably, the voting ensemble classifier proved to be the most effective, when applied to the first small dataset, achieving an impressive accuracy up to 99.56%. The second stacking ensemble classifier proved to be the most effective, when applied to the second large dataset, achieving an accuracy up to 85.6%.

## Introduction

Selecting the optimal crop is a cornerstone for successful agriculture. Detailed analysis and classification based on soil composition, water availability, and prevailing weather conditions are crucial for making informed cultivation decisions, particularly in the face of the unprecedented shifts in our global climate. A substantial body of research highlights the dynamic nature of soil quality and environmental conditions, both of which have profound implications for crop development^1^. Unanticipated climatic fluctuations have wrought significant harm upon the agricultural sector. Such sudden changes have led to diminished crop yields, plunging numerous farmers into precarious situations due to food shortages. This not only imperils individual livelihoods, but also exacerbates food scarcity at a national level.

Therefore, it is of utmost importance to continuously monitor and analyze these variables, as doing so can enhance the productivity of various crops. The agricultural practices and on-farm activities require a significant shift in approach. They need to be more agile and intelligent, adapting proactively to the challenges posed by climate change, thereby minimizing potential crop losses. Moreover, farmers must be armed with comprehensive information, empowering them to discern the crops best suited for specific soil types and conditioned to thrive under particular weather patterns.

Machine Learning (ML) models, when coupled with recommendation systems, have the potential to provide farmers with critical insights into soil conditions, prevailing weather patterns, and the suitability of specific crops for given terrains^1^. At the core of smart agriculture, recommendation systems can be utilized. These systems employ data filtering mechanisms to guide users toward items that align with their preferences. They have found applicability across diverse domains, ranging from movies, consumer products, research articles, and books to healthcare, driving enhancements in performance and user experience^2,3^.

Within the agricultural realm, Crop Selection (CS) is a paramount determinant of the eventual yield^4^. Thus, a crop recommendation system assumes significant importance, aiding farmers by suggesting optimal crops based on myriad factors such as soil attributes and prevailing climatic conditions^5,6^. A recent study examined the application of advanced ML algorithms for crop recommendations^7^. Of the four algorithms evaluated in the study, Random Forest (RF) classifier emerged as the most accurate, boasting a precision of 98.9%. XGBoost closely followed with an accuracy of 98.2%. On the other hand, Logistic Regression (LR) and Decision Tree (DT) models yielded accuracies of 95.6% and 95.3%, respectively.

In the era of digital transformation, the convergence of data analytics and Internet of Things (IoT) is promising for achieving significant advancements in agricultural productivity. IoT systems are expected to play a pivotal role in smart agriculture. They are capable of aggregating pivotal metrics, such as soil moisture, ambient temperature, humidity levels, and pH values. These parameters, collated from a myriad of sensors, are then fed to ML models, thereby enabling farmers to make more informed decisions^8–10^. A notable study presented an integrated system harnessing the capabilities of both IoT and ML, tailored for robust soil analysis^11,12^. This system relied on a suite of sensors for data acquisition and it leveraged multiple ML algorithms, including Support Vector Machines (SVM), LR, RF, Naïve Bayes (NB), DT, and XGBoost classifiers to process and interpret the amassed data.

In this research work, we introduce feature fusion with an ensemble classifier designed to enhance crop yield productivity within the framework of a crop recommendation system. Feature fusion with ensemble algorithms has garnered attention and acclaim for its inherent capacity to enhance predictive outcomes, while simultaneously mitigating the challenges of overfitting and improving accuracy. Their adaptability ensures that they can effectively address a wide spectrum of tasks^13^. This paper examines three key methods within the ensemble algorithms paradigm. They are specifically designed for the nuanced task of crop classification. Numerous factors influence crop production, and among the myriad determinants, this study emphasizes the pivotal roles of soil and climatic conditions. By utilizing these factors, we aim to accurately classify and recommend crop types that align harmoniously with the prevailing environmental factors.

### Motivation

Every crop thrives under a specific balance of soil nutrients, which is instrumental for optimal yield. Traditionally, farmers, lacking precise knowledge of which crops are naturally best suited to their soil types, resort to using fertilizers to supplement the soil nutrient content. While fertilizers can indeed enhance crop growth, their excessive use can lead to environmental degradation. Consequently, making informed crop choices based on the inherent nutrient profile of the soil can minimize the need for fertilization.

### Problem statement

Recent erratic shifts in climate patterns have posed additional challenges for agriculture. These unpredictable variations have led to alterations in optimal sowing periods, resulting in a detrimental impact on crop yields. Thus, there is an urgent need for a crop selection and recommendation system that can mitigate the adverse effects of these climatic changes on agriculture. The ML models have shown promise in the field of agricultural crop management and classification. However, these models often suffer from issues such as overfitting and high computational time. Therefore, further enhancements to ML models are required to address these problems.

### Objective and contributions

The primary objective of this paper is to outline strategies for crop recommendation that aim to mitigate the adverse effects of climate change. This is achieved by deploying a novel crop recommendation system that leverages three methodologies of the stacking ensemble classifier and the voting ensemble classifier. These classifiers play a crucial role in guiding farmers toward crop choices that are most compatible with specific weather conditions and soil characteristics. Additionally, these models solve the problem of overfitting and enhance the computational efficiency.

The salient contributions of this paper can be itemized as follows:

- Proposal of a feature fusion scheme by using three types of feature extraction and concatenating the extracted features to produce the data to be entered into the stacking ensemble classifier.
- Proposal of an innovative crop recommendation system, which is underpinned by three methods of the stacking ensemble classifier.
- Comparative performance analysis of all ensemble classifier types, employing tenfold cross-validation with the introduction of a new Empirical Metric (EM).
- Efficacy comparison of traditional ML models with the proposed dual-stacking ensemble models. The comparative analysis is executed on two datasets for verification.

### Paper organization

The structure of this paper is outlined as follows. “Previous basic models” section delves into the foundational underpinnings of ensemble classifiers, offering insights on their evolution and juxtaposing them with antecedent ML models. “Related works" section is dedicated to a thorough review of relevant literature, encapsulating seminal works and recent advancements in the field. In “Proposed crop recommendation system” section, we introduce our novel crop recommendation system, detailing its architecture and the principles guiding its operation. “Experimental study” section provides an empirical exploration, examining the efficacy of our proposed system through rigorous evaluations and performance metrics. “Comparison of ML methods with the proposed models” section presents a nuanced discussion, comparing the performance of traditional ML methodologies with those of the proposed models, and shedding light on the relative merits of each approach. Finally, “Conclusions and future work” section gives the conclusions and some potential directions for future work in the field of crop recommendation systems.

## Previous basic models

Crop recommendation systems depend on various ML models that help to provide farmers with suitable crops for specific lands that have certain characteristics^14^. In this section, various reference models used for comparison with the proposed models are described.

- *Logistic Regression* (*LR*) *classifier*: It is a basic linear model that depends on the logistic function. Using the given dataset relationship trends, it divides the data into distinct classes. The model is easy to implement, very effective at training, and can categorize unfamiliar data records^15^.
- *Naive Bayes* (*NB*) *classifier*: It is based on Bayes’ probability theory. Given the class variable with the use of probability to select the appropriate crop from testing samples according to the highest likelihood, the NB classifier assumes that the value of a specific feature is independent of the value of any other characteristic^16^.
- *Support Vector Machines* (*SVM*) *classifier*: Data is divided into decision surfaces by SVM classifier. The data is further divided into two hyperplane groups by the decision surfaces. The vector that supports the hyperplane is specified by the training points. This hyperplane is utilized in the process of predicting crops^17^.
- *Decision Tree* (*DT*) *classifier*: Based on the tree data structure, the decision tree is a single-tree predictive model. Decision nodes and decision leaves make up a tree. Each split is identified by a target class, which is a crop with leaf input features. According to the application, the DT classifier employs a top-down method by selecting a value for the variable at each stop that divides a group of items most effectively^18^.
- *Stochastic Gradient Descent* (*SGD*) *classifier*: This classifier is a popular choice for training a wide range of ML models. It is different from the traditional Gradient Descent (GD) classifier in derivative calculation of the loss of a single random data point rather than all data points. This makes it faster than the GD classifier^19^.
- *K Nearest Neighbors* (*KNN*) *classifier*: It is a non-complex algorithm that uses the Euclidean distance to calculate distances and predict a suitable crop based on certain similarity measures. The shortest path between training and testing samples is determined by Euclidean geometry. The closest class is selected, and that class is assigned as a cultivable crop^20^.
- *Linear Discriminant Analysis* (*LDA*) *classifier*: LDA is a straightforward classification technique that identifies straight combinations of characteristics to classify into two or more groups. By displaying spaces in greater dimensions and lower dimensions, LDA algorithm separates the classes into two or more groups. Moreover, the independent variables are combined linearly by LDA to determine the highest class separation ratio^21^.
- *Quadratic Discriminant Analysis* (*QDA*) *classifier*: It is the same as the LDA classifier, but it is mostly employed, when it is impossible to expect that the class dispersion metrics are equal. Additionally, it exhibits low performance, when the class-conditional likelihood densities deviate significantly from the ordinary distribution^22^.
- *Multi-Layer Perceptron* (*MLP*) *classifier*: An MLP is a type of feedforward neural network that aids in overcoming the drawbacks of a single perceptron^23^. It can be used to tackle nonlinear problems, and it is especially appropriate for issues involving time series.

### Background on previous ensemble classifiers

**Fig. 1:**
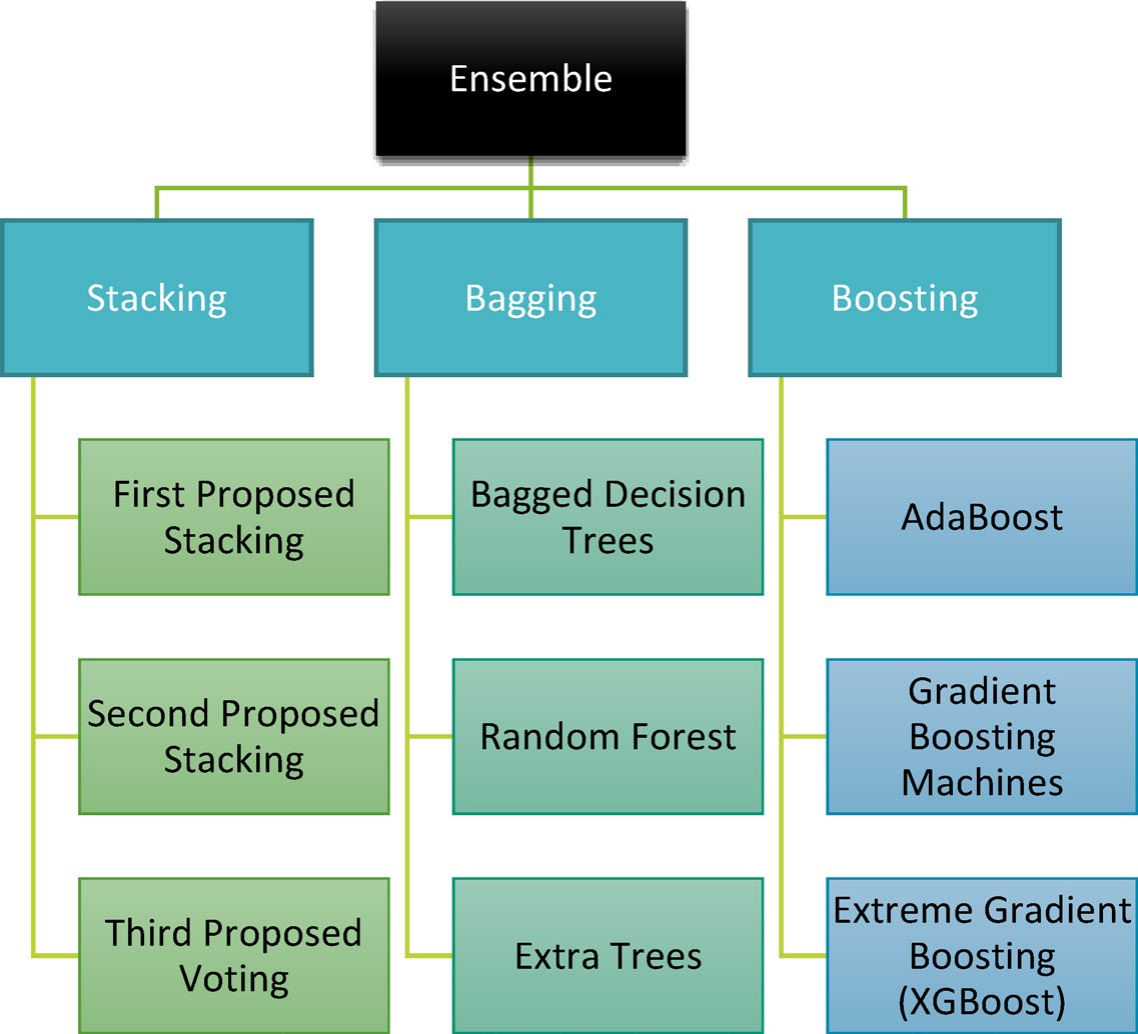
Classification of ensemble algorithms.

Ensemble models have become a cornerstone in traditional ML, capitalizing on the synergy of multiple models to enhance predictive outcomes. The underlying principle involves training a suite of classifiers and subsequently amalgamating their results, either through a voting mechanism or other combinatorial approaches, to achieve an optimal prediction. While ensemble methods undoubtedly bolster predictive accuracy, they introduce a layer of complexity, necessitating thoughtful design and execution. Ensemble techniques can be broadly categorized into three primary methodologies: bagging, boosting, and stacking^23,24^. A visual representation of these methods is shown in Fig. 1.

**Fig. 2:**
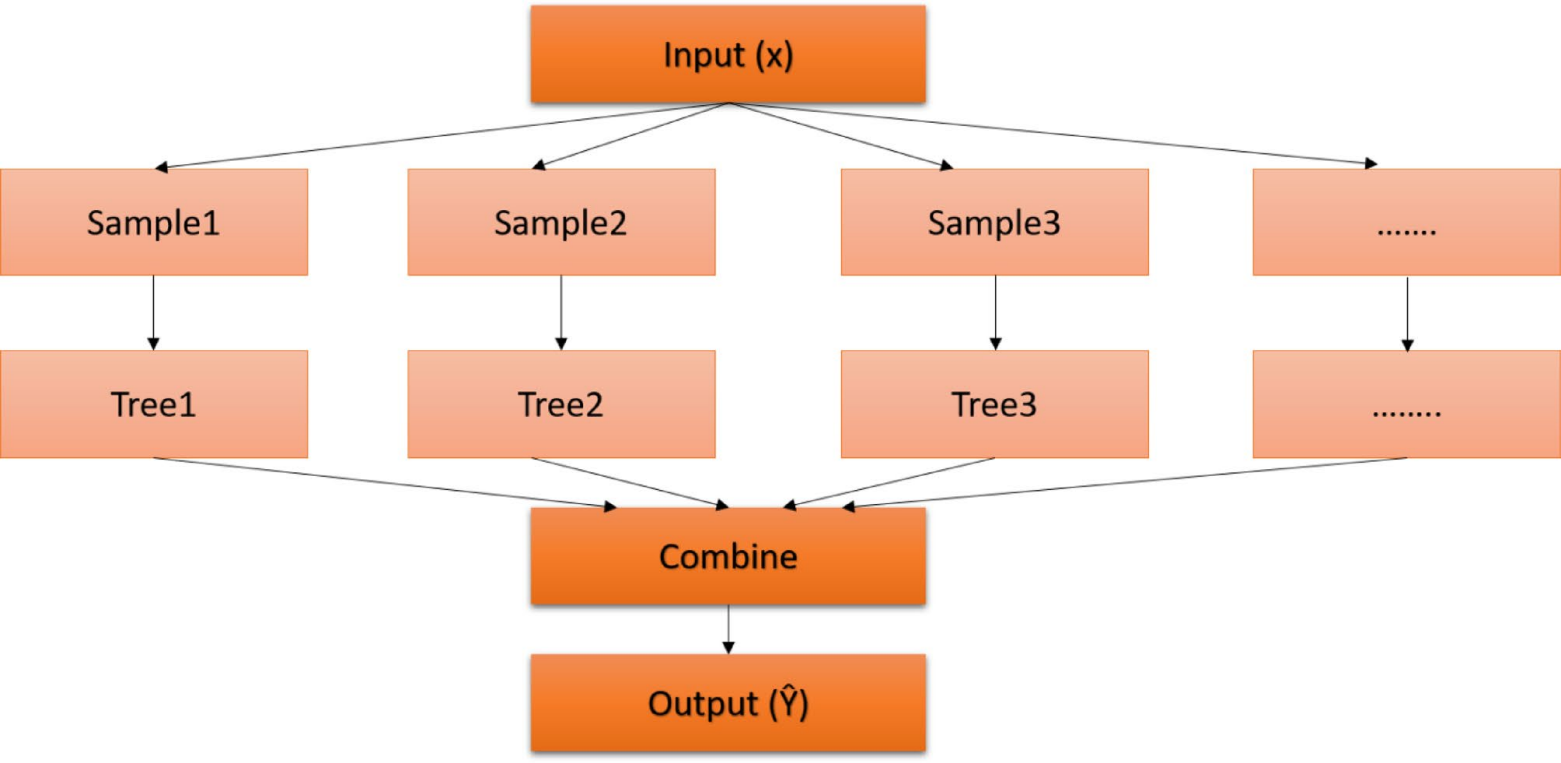
Bagging ensemble model.

**Fig. 3:**
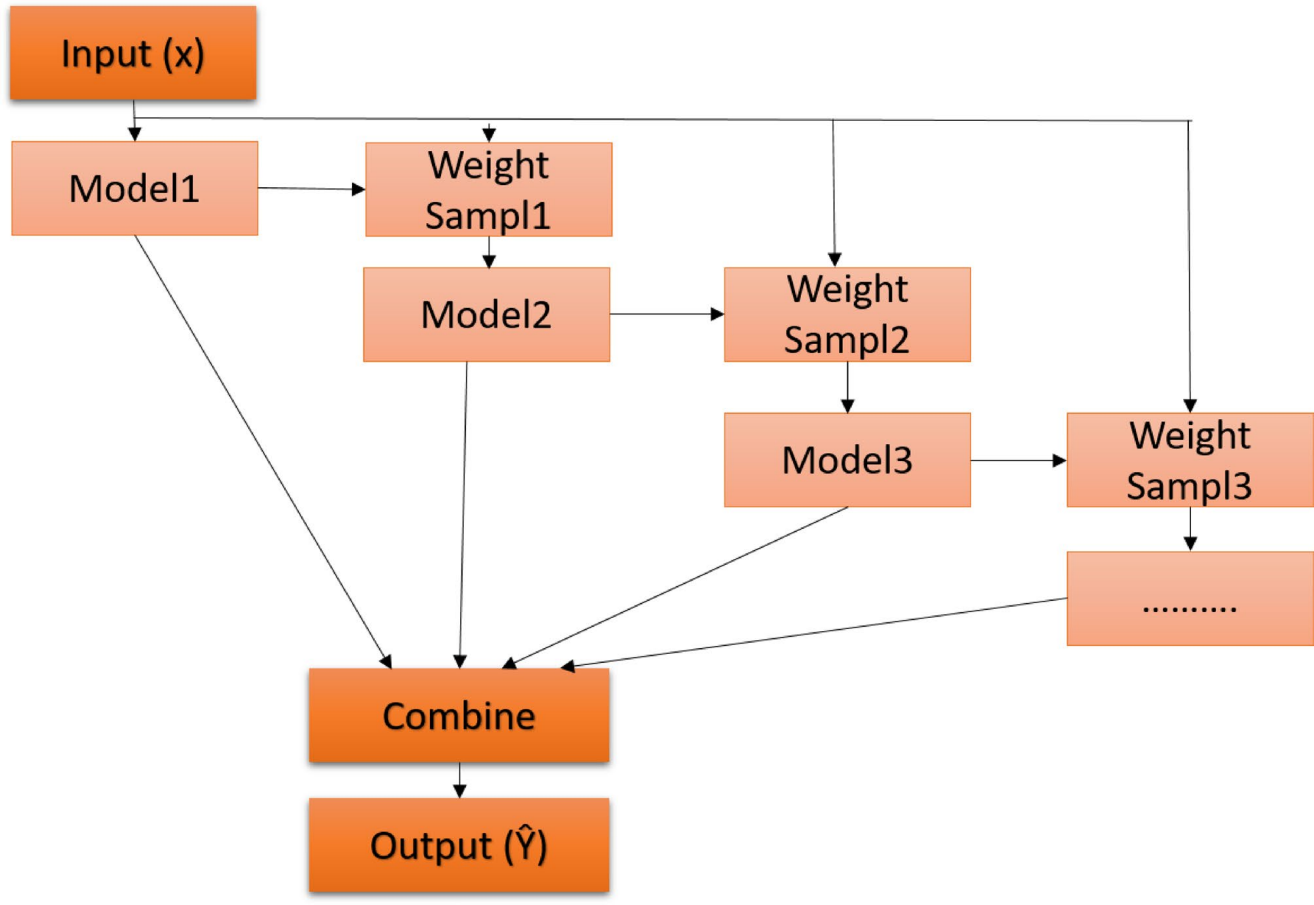
Boosting ensemble model.

- *Bagging model*: Often synonymous with the bootstrap aggregating approach, bagging aims to ensure a balanced representation across the entire dataset. Here, base models operate on ‘bags’ or subsets of the dataset. Notably, each bag retains the dataset original size. The cumulative outputs from all base models are then merged to produce the model final prediction. Figure 2 illustrates the architecture of the bagging ensemble model. Two quintessential examples of this model are Extra Trees (ET) and RF models^25^.
- *Random Forest* (*RF*)*model*: It is a model with relatively simple and easy model with predictive performance compared to other methods. A large number of independent, unpruned decision trees are used in this model. The attributes are selected from the group of the best *n* features, randomly.
- *Extra-Trees* (*ET*) *model*: It is an ensemble model based on trees. In contrast to RF, ET depends on training the base learner using all data rather than bootstrap resampling results. Additionally, ET selects the cutting points for node splitting randomly, rather than calculating them. This provides more randomness than that of the RF classifier.
- *Boosting model*: Boosting is a sequential method that is used to build a new model. The new model depends on the prior one. The new model aims to fix and avoid the mistakes made by its previous models. The result of this process is a final, strong model from weak models. The final model has the weighted mean of all weak models. Gradient Boosting Machines (GBM), Extremely Gradient Boosting Machines (XGBM), and Adaptive boosting (Adaboost) are examples of boosting models^13^. The boosting ensemble model idea is shown in Fig. 3.
- *Adaptive Boosting* (*AdaBoost*) model: It is one of the first useful boosting models. It does not require a large number of hyperparameters, and it runs in polynomial time. When training a new inducer, AdaBoost focuses on occurrences that were previously incorrectly classified. Each instance in the training set is given a weight that determines the level of focus.
- *Gradient Boosting Machines* (*GBM*) *model*: In this type, each inducer model is trained to depend on the previously trained inducers. The primary distinction between GBM and other models is the use of optimization in the function space of GBM. The correlation related to the whole ensemble is maximized with the negative gradient of the loss function by including a learning procedure in which the goal is to construct the base learners.
- *Extreme Gradient Boosting* (*XGBoost*) *model*: It is a faster variant of GBM, as it provides parallel preprocessing at the node level. To lessen overfitting, XGBoost also offers several regularization techniques.

## Related works

By utilizing an ML system, the study of^26^ helped farmers recommend suitable crops for yield based on specific input factors. Temperature and humidity are collected through the DHT11 sensor using the MCU node, and NPK. The pH, and rainfall values are directly fed from the crop dataset. By using the Kodular creator, a mobile application is created and connected to the Firebase cloud platform, and it is intended for farmers’ assistance. The suggested approach gives better results for crop recommendation classifiers with an accuracy of more than 95%.

Another study^27^ presented a machine-learning-enabled system for crop recommendation, aimed at helping farmers make informed decisions to optimize crop yield. It developed a robust ML model including GNB, LR, KNN, DT, RF, and SVM classifiers that support decisions about crop selection. The authors developed a web-based application for farmers to interact with the model. This application shows a practical and user-friendly approach to deploying the system. The GNB classifier achieved a good model accuracy equal to 99%.

The Artificial Intelligence (AI) recommendation system presented in^28^ contributed to the overall improvement in crop harvesting accuracy and quality in precision agriculture. Eight features with seven independent variables, namely temperature, humidity, pH, rainfall, N, P, and K, were included in the dataset that was taken from the Kaggle website. Using the SMOTE data balancing technique, the CatBoosting strategy yielded the best results, with accuracy values of 99.5129, F-measure of 0.9916, Precision of 0.9918, and Kappa of 0.8870.

The authors of^29^ achieved optimal productivity through strategic crop cultivation. This approach not only identifies weeds, but also offered specific herbicide recommendations. Additionally, the authors explored several algorithms tailored for crop recommendation, while providing an intricate cost analysis for crop cultivation, encompassing factors like fertilizers, seeds, manures, and labor costs.

The authors of^30^ focused on leveraging deep learning to enhance crop recommendation systems in agriculture. They used advanced deep learning models, such as Long Short-Term Memory (LSTM), Bidirectional LSTM, and Transformers, for more accurate and dynamic crop recommendation. The Transformer Encoding Model (TEM) demonstrated the ability to handle complex agricultural data and achieved a high accuracy of 98.87%.

In^31^, the dataset was preprocessed after being collected from Bangladesh pertinent research institutions. Utilizing an ensemble model known as K-nearest neighbors Random forest ridge Regression (KRR), the study worked on accurately forecasting the yield of three different crops: wheat, potato, and rice. KRR was compared with five conventional ML methods (RF, CatBoost, ridge regression, Naïve Bayes, and support vector regression). In comparison to the examined ML methods, KRR exhibits minimal errors and achieves a maximum *R*^2^ score of 99% in addition to a minimum Mean Square Error (MSE) of 0.009, according to the evaluation criteria. A crop recommender system that suggests the best crops to grow on a certain land was also introduced.

By selecting the best crops for certain soil and geographic conditions, the authors of^32^ aimed to create an efficient crop recommendation system that lowers losses. An Adaptive and Attention-based Hybrid Network (AAHNet) paradigm was employed in the presented system. For deep feature extraction, this model incorporates a serial cascaded network, which combines a 1D Convolutional Neural Network (1DCNN) with an autoencoder. A Modified Movement Territory of Fire Hawk Optimizer (MMTFHO) method is then used to optimize the AAHNet parameters and choose the best features from the retrieved data.

AgroXAI, an explainable AI-driven crop recommendation system created for Agriculture 4.0, was introduced in^33^. By promoting crop diversity, the system aims to address the challenges facing agriculture, including population growth, climate change, and land degradation. To provide both local and global explanations for ML model decisions, the system integrates several XAI techniques, including SHAP, counterfactual, LIME, and ELI5. For crop categorization, the study employed several ML models, including MLP, LightGBM (LGBM), SVM, RF, DT, and KNN. The RF model demonstrated the best accuracy of 99.24%.

The two graph-based crop recommendation systems, Graph Convolutional Network (GCN) and Graph Neural Network GNN, were presented and compared in^34^. Based on factors such as temperature, humidity, soil pH, rainfall, and levels of nitrogen, potassium, and phosphorus, the best crop for a particular season is selected. The study used data preprocessing to generate a graph. For GCN and GNN models, the F1-score, recall, accuracy, and precision are used to evaluate the efficacy of crop recommendation. *K*-fold cross-validation is another technique used to guarantee generalizability and avoid overfitting. The study demonstrated that GCN outperforms GNN in scenarios requiring more extensive feature interactions, as it captures localized connections in the feature graph. Furthermore, the accuracy offered by the GCN model was almost 98%.

The authors of^35^ examined crop recommendation performance using seven different ML methods. The suggested system utilizes several characteristics, including information on climate and soil composition, to accurately predict which crops will be best suited for a specific area. By training and testing models with various configurations of ML algorithms, the system introduced in the paper has achieved high accuracy through an exhaustive examination of a large historical dataset. Additionally, the paper introduced an accuracy of over 95% for all models.

Finally, in^36^, the Synthetic Minority Oversampling Technique (SMOTE) was used to balance the dataset for the proposed model. The authors implemented 13 different classifiers, namely Logistic Regression (LR), DT, KNN, SVC, RF, GB, Bagged Tree (BT), XGB, AdaBoost, CatBoost, Histogram-based Gradient Boosting (HGB), Stochastic Gradient Descent Classifier (SGDC), and Multinomial Naive Bayes (MNB) classifiers on a large dataset of 246,091 sample records with 37 different crops. The SGDC approach yields better results for crop recommendation classifiers, achieving an accuracy of 100%, but with low sensitivity and specificity. The study also discussed several intriguing future concepts and issues facing the agriculture sector. All related works are summarized and compared in Table 1.

**Table 1:** Summary of related works.

Paper	Contributions	Dataset	Data preprocessing	ML algorithms	Advantages	Disadvantages
^26^	Utilization of sensors to collect temperature and humidity with ML models for a crop recommendation system and a mobile application for farmers	Crop dataset with features (N, P, K, T, humidity, and rainfall)	Feature selection (wrapper method) Data splitting	Various models, such as KNN, DT, SVC, NB, RF, Gboost, ET, and XGB	Crop recommendation prototype, and user-friendly mobile application	The limited number of features makes the system inflexible, neglecting overfitting and calculation time
^27^	ML-enabled system for crop recommendation to assist farmers in making informed decisions for optimal crop yield	Collected parameters (NPK values, pH, temperature, humidity, and rainfall)	Handling missing values, and removal of duplicate features	Various models such as GNB, LR, KNN, DT, RF, and SVM	Providing high accuracy, and a user-friendly framework	The limited dataset used, neglecting overfitting and calculation time
^28^	Application of feature selection and feature extraction based on AI, and a variety of ML algorithms for crop recommendation	Twenty-two crop dataset with features N, P, K, T, humidity, pH, and rainfall	Feature extraction, and feature selection	SMOTE data balancing technique and 14 different ML models	Enhancing accuracy by using feature extraction, selection, and data balancing techniques, and utilization of large numbers of ML models	Need for a friendly framework for farmers, neglecting overfitting and calculation time
^29^	Proposal of four modules, namely recommendations, weed identification, pesticide recommendation, and crop cost estimation	Crop dataset, soil dataset, weed dataset, pest dataset, and crop name dataset	Outlier detection and treatment, conversion of categorical data to numerical values, and data splitting	Multiple models with tuning hyperparameters, such as KNN NB, LR, SVM, RF, Adaboost, Gboost, and XGB	High crop productivity, low cost, offered specific herbicide recommendation, friendly framework	Accuracy needs to be enhanced, and overfitting and calculation time are neglected
^30^	Utilization of deep learning to enhance crop recommendation systems in agriculture	Dataset including soil nutrients (NPK, pH), and weather data (temperature, rainfall, humidity)	Cleaned errors and missing values, removal of outlier data formatting	Long Short-Term Memory (LSTM), and Bidirectional LSTM (BiLSTM)	Utilization of deep learning models, and friendly frameworks	Complex computations, neglecting overfitting and calculation times, and the need for comparison with traditional ML algorithms
^31^	Design of a recommender system by using a blending ensemble model to suggest suitable crops for a specific land area for cultivation in the next season	Self-generated dataset with 7,000 records for five crops (Aus rice, Aman rice, Boro rice, potato, and wheat)	Handling missing values, wrong formats, wrong data, data integration and reduction, and data splitting	KNN classifier, RF classifier, and KRR model	Self-generated dataset, reducing overfitting, and flexibility of learning from the dataset	Complex model, neglecting calculation time, and need for a friendly framework
^32^	Utilization of a deep learning framework for crop recommendation, with advanced feature extraction, optimization, and hybrid neural network models	Three crop recommendation datasets from various places	Deep feature extraction (serial cascaded network combining a 1D CNN with an autoencoder)	AAHNet MMTFHO for optimization	Three datasets for evaluation, integrated approach for feature extraction, utilization of DL models, and utilization of an optimization model	Complex model, neglecting overfitting and calculation time, need to enhance accuracy, and need for a friendly framework
^33^	Proposal of AgroXAI, an edge computing-based explainable crop recommendation system to suggest suitable crops	Dataset containing 22 different crops, 2200 rows of data, and seven features	–	MLP, LightGBM, SVM, RF, DT, and KNN	Utilization of AgroXAI for an explainable crop recommendation system	Need for real-time data gathered by sensors, neglecting overfitting and calculation time, and need for a friendly framework
^34^	Comparison of two graph-based crop recommendation algorithms, GCN and GNN	Crop dataset with features (N, P, K, T, humidity, pH, and rain fall)	Data normalization, scaling, and data splitting	Two graphical models (GCN and GNN)	Model reduces overfitting	Neglecting calculation time, and need for a friendly framework
^35^	Examination of crop recommendation performance using seven different ML models	Crop dataset with features (N, P, K, T, humidity, pH, and rainfall)	Data cleaning, removal of outliers, and data splitting	Seven models (LR, DT, RF, KNN, NB, SVM, and neural network)	Application of various configuration parameters for models	Need for real-time data gathered by sensors, neglecting overfitting and calculation time, and need for a friendly framework
^36^	Utilization of SMOTE to balance the dataset with ML models for the crop recommendation system	246,091 sample records with 37 different crops	Cleaning of irrelevant fields, and data splitting	Utilization of 13 classifiers (LR, DT, KNN, SVC, RF, Gradient Boost, Bagged (GBB), XGB, AdaBoost, CatBoost, HGB, SGD, and MNB)	Utilization of a large number of classifiers, utilization of a large dataset with large numbers of crops, and user-friendly mobile application	Neglecting overfitting and calculation time

## Proposed crop recommendation system

In this study, we introduce feature fusion by using three types of feature extraction, which are applied with three novel models based on the stacking ensemble technique to classify and provide crop recommendations. Additionally, we explore two other ensemble algorithm methods, examining their respective models and comparing them with our proposed models.

The first method we explore is the Boosting model. It begins by training on a subset of the dataset. After an initial round of predictions, it assigns greater weights to correctly-predicted data points. Subsequent models then focus on refining predictions by addressing previous inaccuracies. We perform experiments with three distinct Boosting classifiers, namely GBM, XGBM, and Adaboost, assessing their efficacy in crop recommendations.

The second method under examination is the Bagging model. In this method, multiple datasets are derived from the original training set, with each dataset being used to train a separate base model. These base models may involve resampling observations. The culmination of this method involves aggregating predictions from all base models. Notably, the Bagging method predominantly employs a single type of base models. In our investigation, we evaluated three classifiers associated with the Bagging model: ET, RF, and Bagged Decision Trees (BDT) classifiers.

**Fig. 4:**
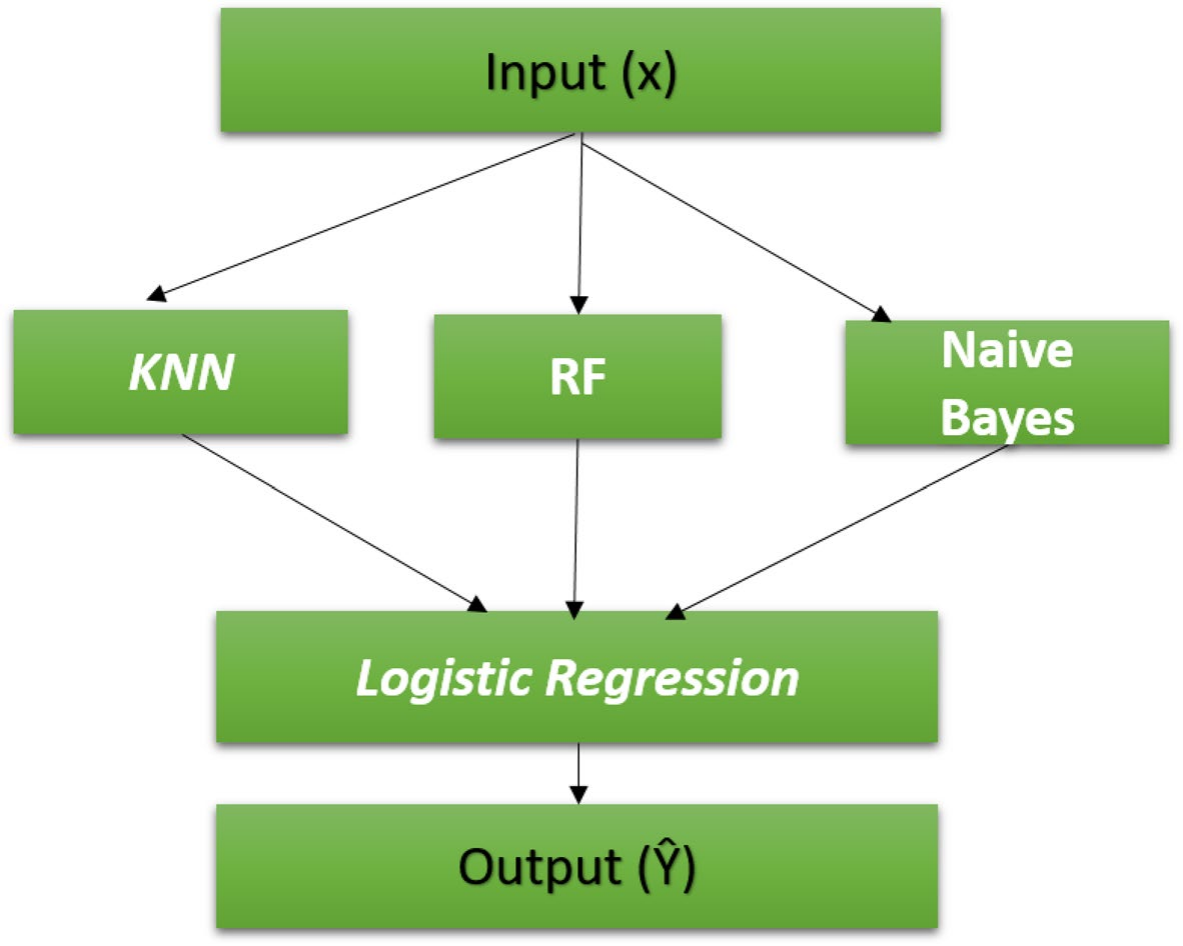
First proposed stacking model.

The models we propose are based on the stacking ensemble technique with feature fusion. The stacking is a technique that produces a combination of multiple models (classification or regression) and a meta-model (meta-classifier or meta-regression)^37^. Stacking has a first level that uses multiple models and a second level that uses the meta-model, which sets it apart from the fundamental ensemble methods. Training of all base models at the first level initially extracts the stacking features. The basic models used in this level are different in their methods of dataset classification, and the training processes for these basic models are implemented in a parallel way. The training stacking features are used to train the second-level model to predict the final output, thereby achieving the best accuracy. In the first level of the stacking classifier, each base model operates by partitioning the training dataset into '*n*' distinct segments. Subsequently, '*n*-1' of these segments are used to train the base model. The base model then generates predictions for the remaining part. Once this is completed, the base model is re-trained using the entire training dataset, and predictions are made on the test dataset. This procedure is repeated for each new base model. Every iteration offers a new set of predictions for both the training and testing datasets. As a culmination of this process, a fresh model is derived from the predictions made on the training dataset, which now serve as features. These features are then fed into the second level, where the meta-model is trained to provide crop recommendations. In the first proposed stacking model, we try to select the most efficient base model that gives high accuracy and low overfitting. So, we use KNN, RF, and NB classifiers, as shown in Fig. 4.

**Fig. 5:**
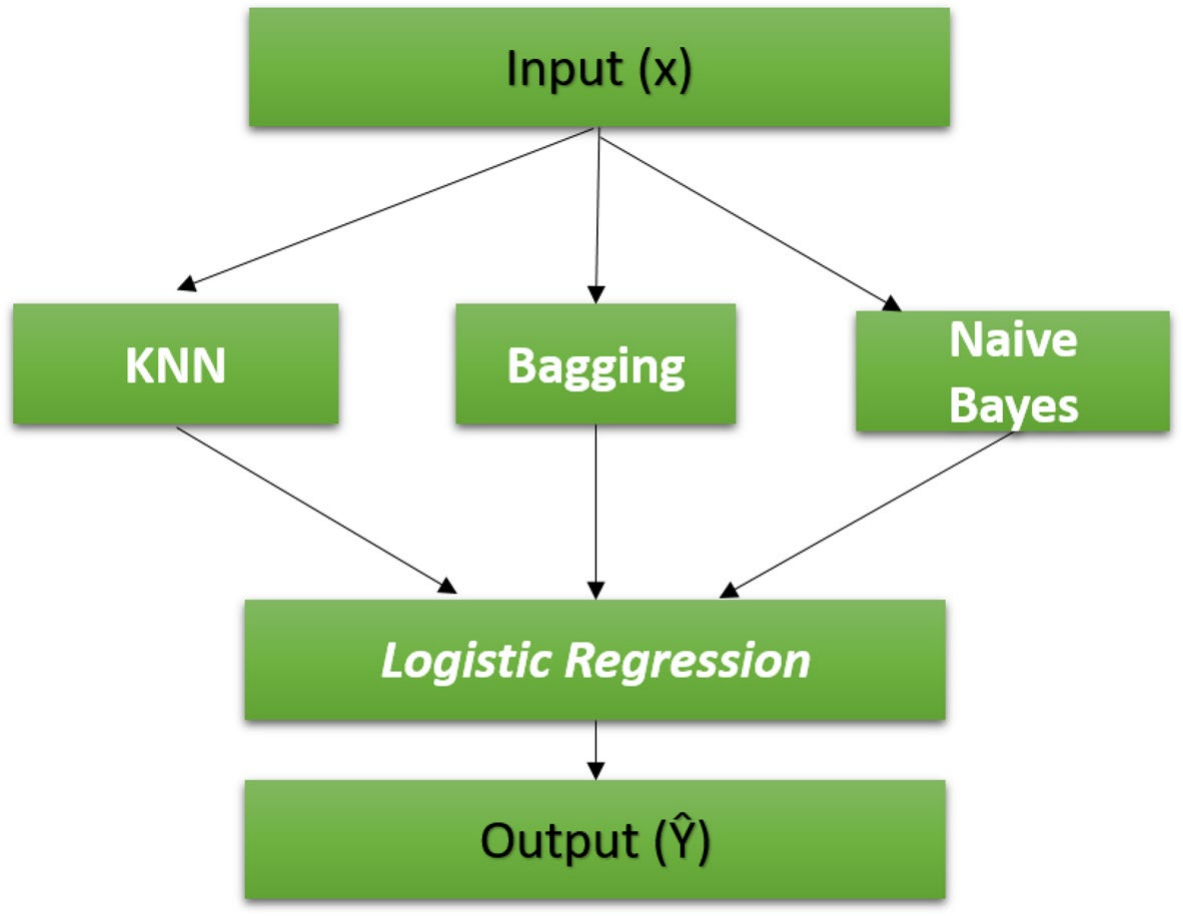
Second proposed stacking model.

The second proposed stacking model gives high accuracy, low overfitting, and low computational time. In this model, we use KNN, Bagging, and NB classifiers, as shown in Fig. 5.

**Fig. 6:**
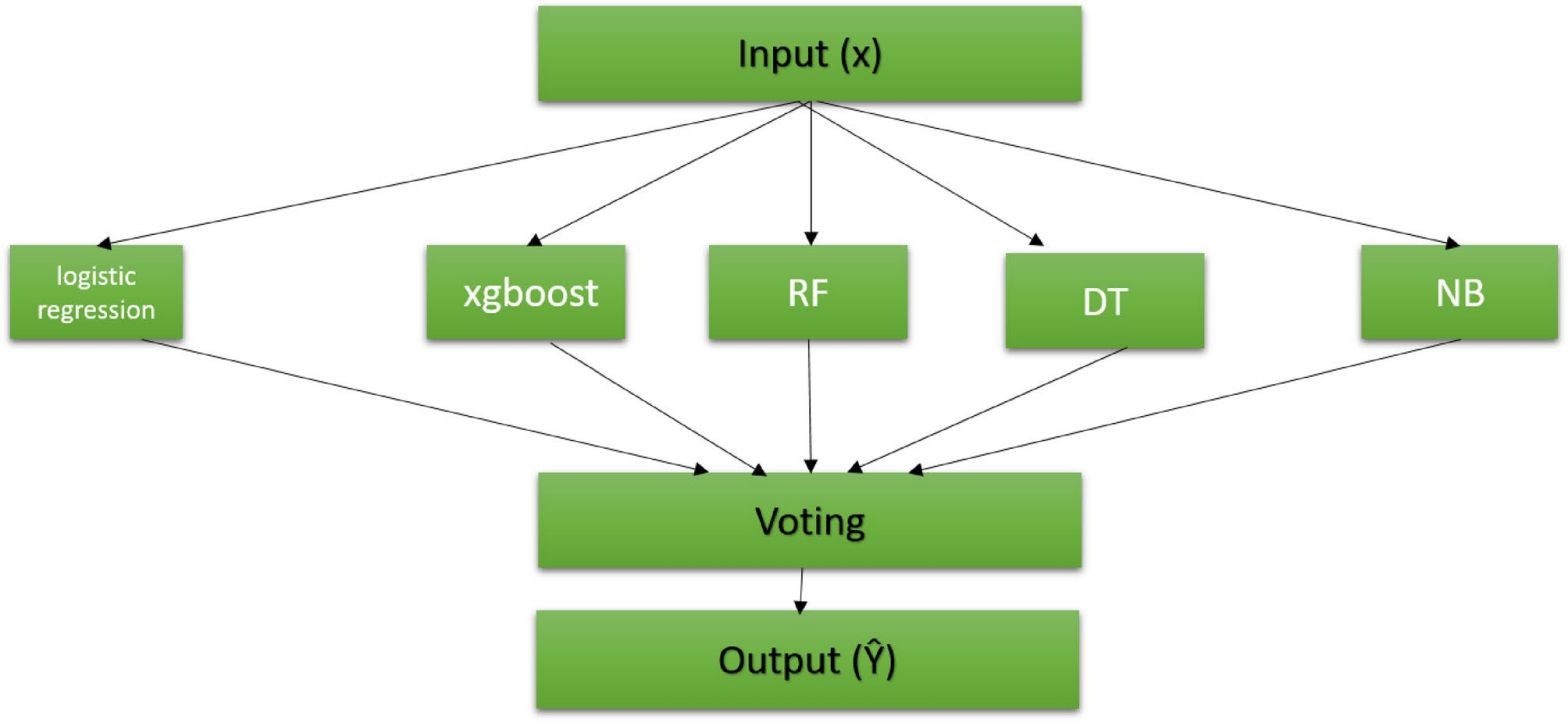
Third proposed voting model.

The third proposed model is based on a voting classifier. It depends on the principle of collective decision-making. It also has two levels and differs from the previous stacking models in that the second level is where the voting process is implemented. Multiple models are constructed, each rendering its prediction, colloquially termed its “vote”. The outcome or decision that garners most votes from these models is then selected as the final output, as shown in Fig. 6. We have conducted a comparative analysis between our three proposed classifiers and other pre-existing ensemble models to evaluate their performance and efficacy.

## Experimental study

**Fig. 7:**
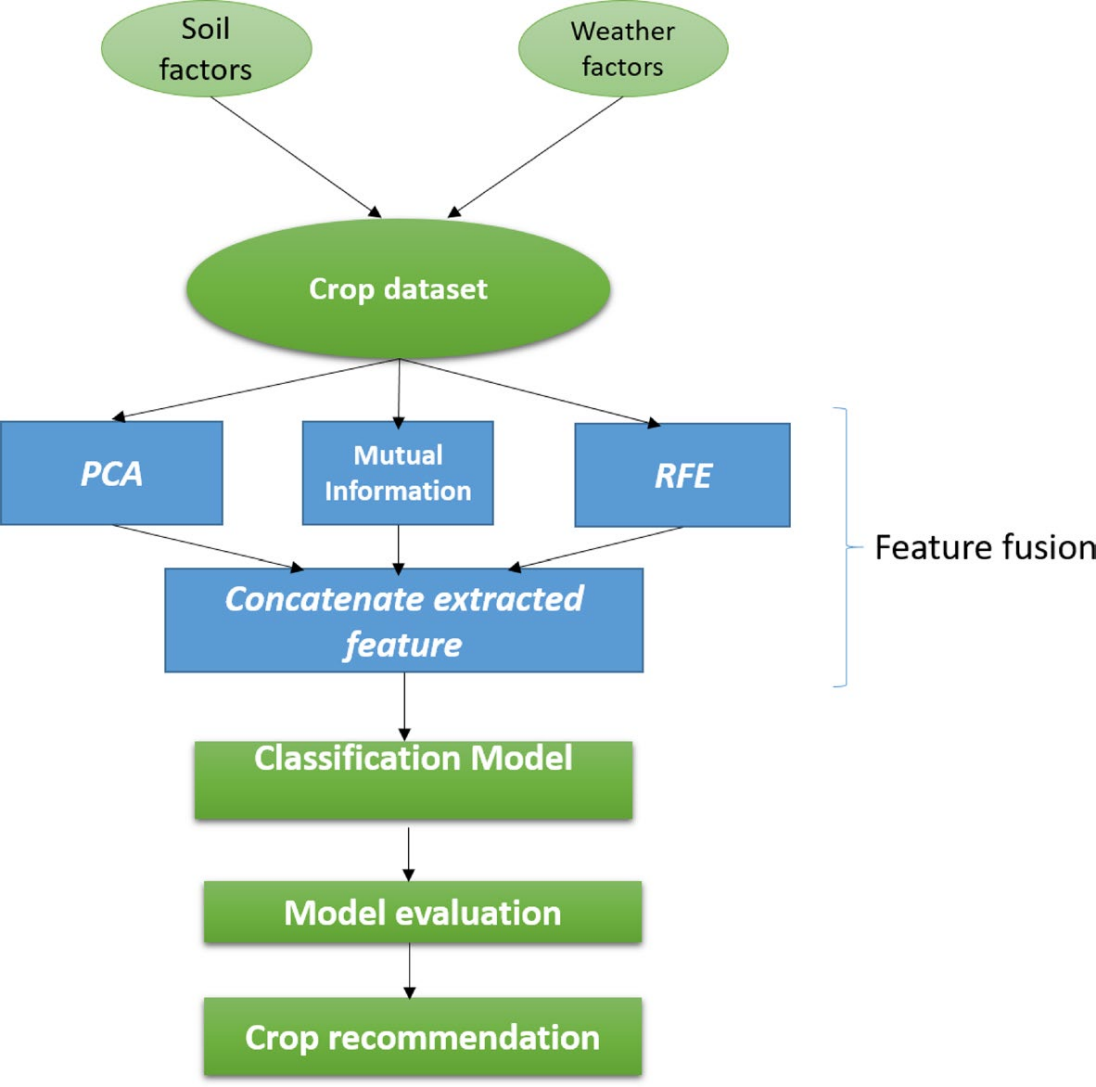
Flowchart of the crop recommendation system.

In our research endeavor, we utilized the Python language and the sklearn library, executed within the Spyder development environment, to facilitate diverse algorithmic operations ranging from feature extraction to regression and classification. The software runs on a laptop with a Core i5 processor and utilizes hardware resources, including 8 GB RAM. Within the coding structure of our program, we strategically employ three classifier models with feature fusion. Three types of feature extraction are employed to extract and concatenate features, resulting in a fused feature set that is then fed into classification models based on the stacking ensemble paradigm. These models predict optimal crop choices by offering recommendations based on intricate interactions between soil and climatic variables. Figure 7 provides a graphical representation of the crop recommendation system flow.

The initial classifier model, anchored in the stacking ensemble approach, seamlessly integrates three foundational models for primary-level classification, namely KNN, RF, and NB. At the second level, the LR model is employed to consolidate inputs and derive the final predictive output.

In the second classifier model of the stacking ensemble approach, three foundational models, KNN, Bagging, and NB, are combined at the primary level. Similarly, the LR model is employed at the second level to consolidate the inputs and derive the final predictive output.

Our subsequent proposal revolves around the voting ensemble technique. Within the implementation, we incorporate five foundational models for initial-tier classification. These are LR, XGBoost, RF, DT, and GNB. Once these models are trained, their collective outputs are channeled into the subsequent level. Here, an aggregation process evaluates the outputs, and through a majority voting mechanism, the model with the predominant vote determines the final result.

### Dataset

**Fig. 8:**
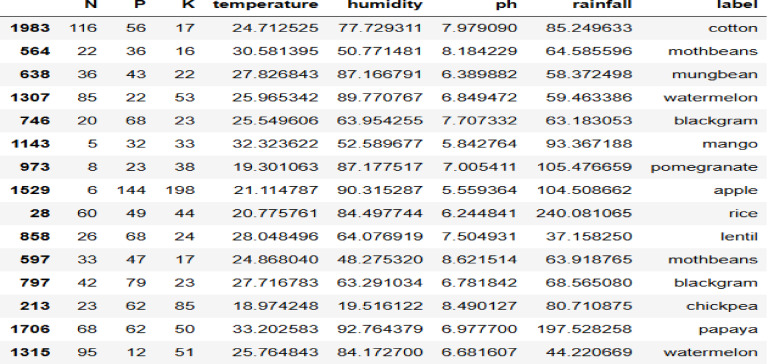
Samples of the first crop dataset.

In our research, we employed two types of datasets for validation and verification of our proposed models. The first dataset comprises 2200 records, each representing unique soil and weather conditions essential for cultivating various crops, as shown in Fig. 8. Pertinent soil nutrients detailed in the dataset include pH value, Potassium (K), Phosphorus (P), and Nitrogen (N). Concurrently, the climatic parameters encapsulated are humidity, temperature, and rainfall, each of which plays a crucial role in influencing crop yield and health. This dataset was sourced from the widely recognized open-source platform, Kaggle. Additionally, it is enriched with information on twenty-two diverse crops, encompassing staples like rice and maize, as well as fruits such as apples and mangoes.

**Fig. 9:**
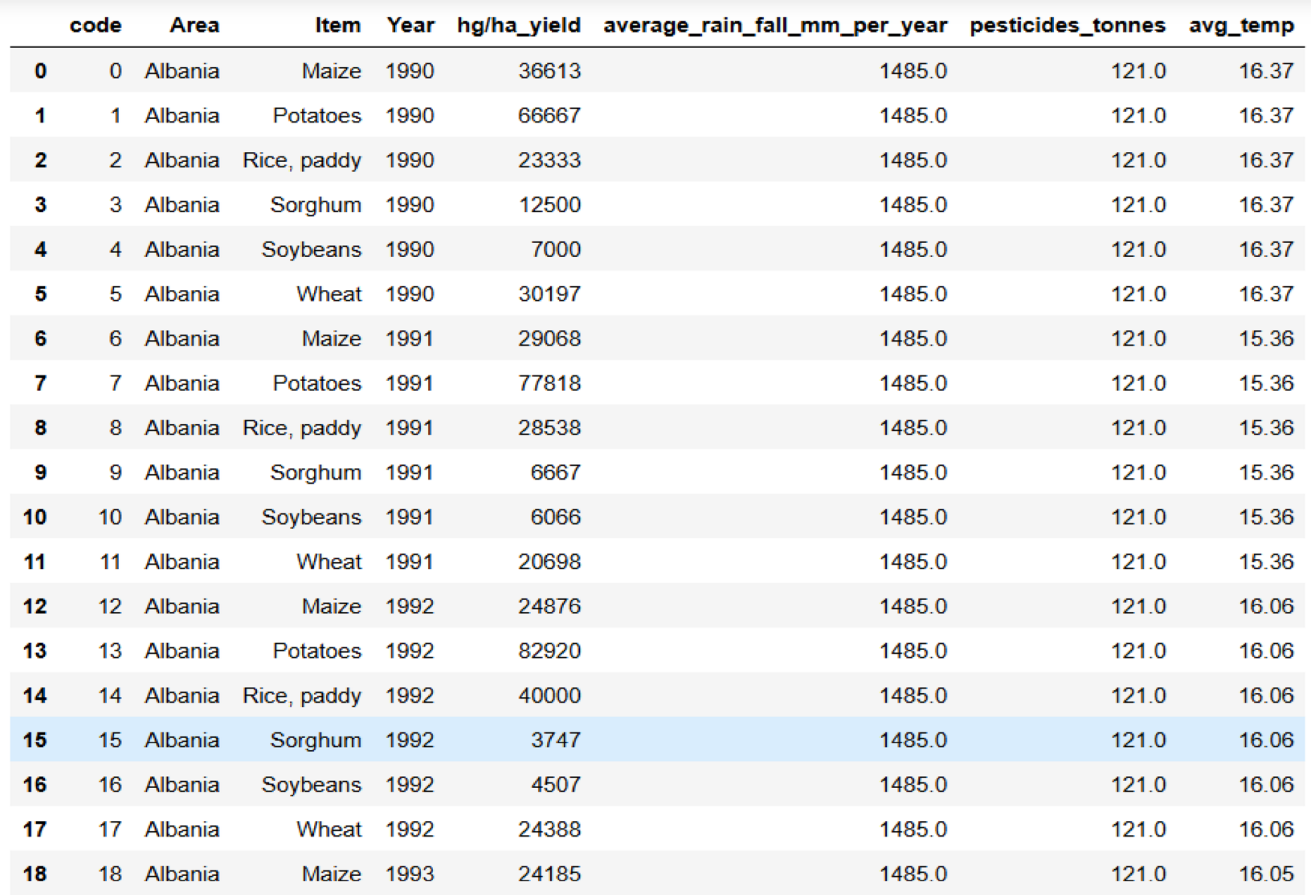
Samples of second crop dataset.

The second dataset is a large dataset containing 28,242 records sourced from the Food and Agriculture Organization (FAO), as shown in Fig. 9. Key features of this dataset include country code, area, item, year, hectare yield, average annual rainfall, pesticide usage in tons, and average temperature. It is enriched with information on various crops, including rice, wheat, maize, and others.

### Dataset preprocessing

#### Data splitting

In our analysis, we adopted two distinct strategies for partitioning the dataset to ensure the reliability and robustness of our results:

- *Method one*: The primary approach involved a straightforward split, allocating 80% of the dataset for training and the remaining 20% for testing. By employing this methodology, we gauged various metrics, including training accuracy, testing accuracy, precision, recall, and the F1 score, across all models under consideration. A noteworthy observation was the variability in results for certain models when executed multiple times. This inconsistency is attributed to the inherent randomness in selecting different training and testing sets. To achieve more consistent results, we turned to the cross-validation technique as an alternative approach.
- *Method two*: We employed the cross-validation technique, specifically tenfold cross-validation. This method leverages the data subsets from the initial 80/20 split of the first approach. In a tenfold CV, the training set, which constitutes 80% of the dataset, is further divided into ten subsets. During each iteration, one subset serves as the validation set, while the remaining nine form the training set. This iterative process ensures that each subset gets used as a validation set, once. As a result, ten distinct training accuracy scores are obtained, and their average offers a more stable representation of model performance. This procedure is mirrored on the testing set (the 20% portion) to yield an average testing accuracy, thus providing a comprehensive understanding of the model capabilities.

#### Feature fusion

In our analysis, we propose a feature fusion approach that utilizes three types of feature extraction and concatenates the extracted features for classification, thereby enhancing our results. The three feature extraction techniques are:

- *Principal component analysis* (*PCA*): It is a practical statistical signal processing approach that reduces the dimensionality of datasets for compression, pattern detection, and data interpretation^38^. It achieves this by identifying new axes that reveal the most variations in the data, known as primary components. These elements are a potent tool for dimensionality reduction, since they are orthogonal to one another, which means that they are uncorrelated.
- *Mutual information*: It measures the degree to which two variables are dependent on one another, primarily indicating their correlation^38^. The stronger the correlation is, the more the information between the variables. The so-called feature selection method, based on maximum correlation and minimum redundancy, is used in data processing to reduce data redundancy by eliminating features with low mutual information and selecting features with high mutual information with the target variable, thereby increasing the algorithm predictive ability.
- *Recursive feature elimination* (*RFE*): This algorithm for feature selection works by progressively removing features that are not important. The RFE is now a widely used feature selection technique in many ML applications, including prediction and classification. It can be used to remove traits that are not important, improving classification accuracy. It can be used to reduce the model size. Applications such as deploying ML algorithms on mobile devices greatly benefit from this issue^39^.

#### Explainable AI

Crop categorization systems are validated through the use of explainable artificial intelligence (XAI). It enables us to pinpoint the crucial elements in crop data that affect choices. By lessening the impact of noisy features, it verifies the accuracy of predictions^40^. It also provides comprehensive explanations of how the model makes predictions for crop recommendations, which features are most important, and why certain predictions might be incorrect. The explanations will be both visual and textual, making it easier to understand and communicate the model decision-making process.

### Evaluation metrics

In this section, we provide an overview of the evaluation metrics mentioned and used, particularly in the classification process. The effectiveness of the models has been evaluated using several performance metrics depending on important parameters such as True Positive (TP), True Negative (TN), False Positive (FP), and False Negative (FN) rates. The most frequently-used metrics are Accuracy, Precision, Recall, and F1 score, which are stated in Eqs. (1–9).

- *Accuracy*: It is the statistical indicator that is most frequently utilized. Its value is between 0 and 1. A close value to 1 indicates accurate prediction. There are training Accuracy (NA) and Test Accuracy (TA).
- *Precision* (*Pr*): It is calculated by dividing the number real positive predictions by the number of all positive forecasts. Its range is from 0 to 1, with values close to 1 indicating fewer false positive predictions.
- *Recall* (*Re*): It is employed to examine uncertainty, when using model prediction. A value close to 1 indicates few erroneous negative predictions. Its value is between 0 and 1.
- *F1 score* (*F1*): It is referred to as the Precision and Recall weighted harmonic mean. It implies the accuracy of the measurement test. Its value is from 0 to 1, and when the value reaches 1, it means better Precision and Recall.
- *The Cohen’s Kappa* (*K*): It can be used to measure the degree to which two or more raters can evaluate, and rate behavior. Its value is determined through the confusion matrix. Kappa score can be computed for binary or multiclass problems, and its value is between -1 and 1.
- *Hamming Loss* (*HL*): It gives the Hamming distance between two sets of samples. In multiclass classification, the Hamming loss corresponds to the Hamming distance between true and predicted values, and it is always between 0 and 1.
- *Jaccard Score* (*J*): It is a statistic measure used to represent the similarities between sample sets, and it is also known as the Jaccard index or Jaccard similarity coefficient. It is used to emphasize the similarity between finite sample sets. It is formally defined as the size of the intersection divided by the size of the union of the two labeled sets. Its value is between 0 and 1.
- *Matthews correlation coefficient* (*MCC*): It is a statistical metric generally used for binary classification assessment. It ranges between -1 and + 1, and it has a broad consideration of all confusion matrix values.
- *Empirical Metric* (*EM*): It is a proposed metric composed of the absolute values of other metrics regardless of their units to represent a general trend in system performance. It is estimated as the average of all evaluation metrics and the complement of the Hamming loss.
- *Computational time* (*CT*): It refers to the time required for model training and evaluation to determine the recommended crop.
  1 $$Accuracy=\frac{ TP + TN}{TP + FP + FN + TN}$$
  2 $$Precision =\frac{TP}{TP + FP}$$
  3 $$Recall = \frac{TP}{TP + FN}$$
  4 $$F1 score = \frac{2 \times Precision \times Recall}{ Precision + Recall}$$
  5 $$K=\frac{2(TP\times TN-FP \times FN)}{(TP+FP)(TP+FN)(TN+FP)(TN+FN)}$$
  6 $${\it{HL}} = \frac{1}{NL}\mathop \sum \limits_{l = 1}^{L} \mathop \sum \limits_{i = 1}^{N} {{\it{Y}}}_{{\it{i},\text{l}}} \oplus {\hat{\it{Y}}}_{{\it{i},\text{l}}} $$
  7 $${J = \frac{\mid{{\hat{\it{Y}}} \cap Y}\mid}{\mid{{\hat{\it{Y}}} \cup Y}\mid}}$$

where ${\hat{\it{Y}}}$ is the $\text{predicted value}$, and *Y* is the actual value.

8 $$MCC=\frac{\left(TP\times TN\right)-(FP \times FN)}{\sqrt{(TP+FP)(TP+FN)(TN+FP)(TN+FN)}}$$

9 $$\it{EM}=\frac{N{A}+T{A}+Pr+Re+F1+K+HL^{c}+J+MCC}{N}$$

where *N* is the number of all metrics.

### Experimental results

**Fig. 10:**
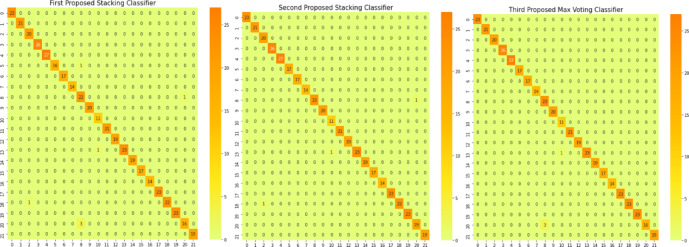
Confusion matrices for the first proposed staking classifier, second proposed staking classifier, and third proposed voting classifier.

**Fig. 11:**
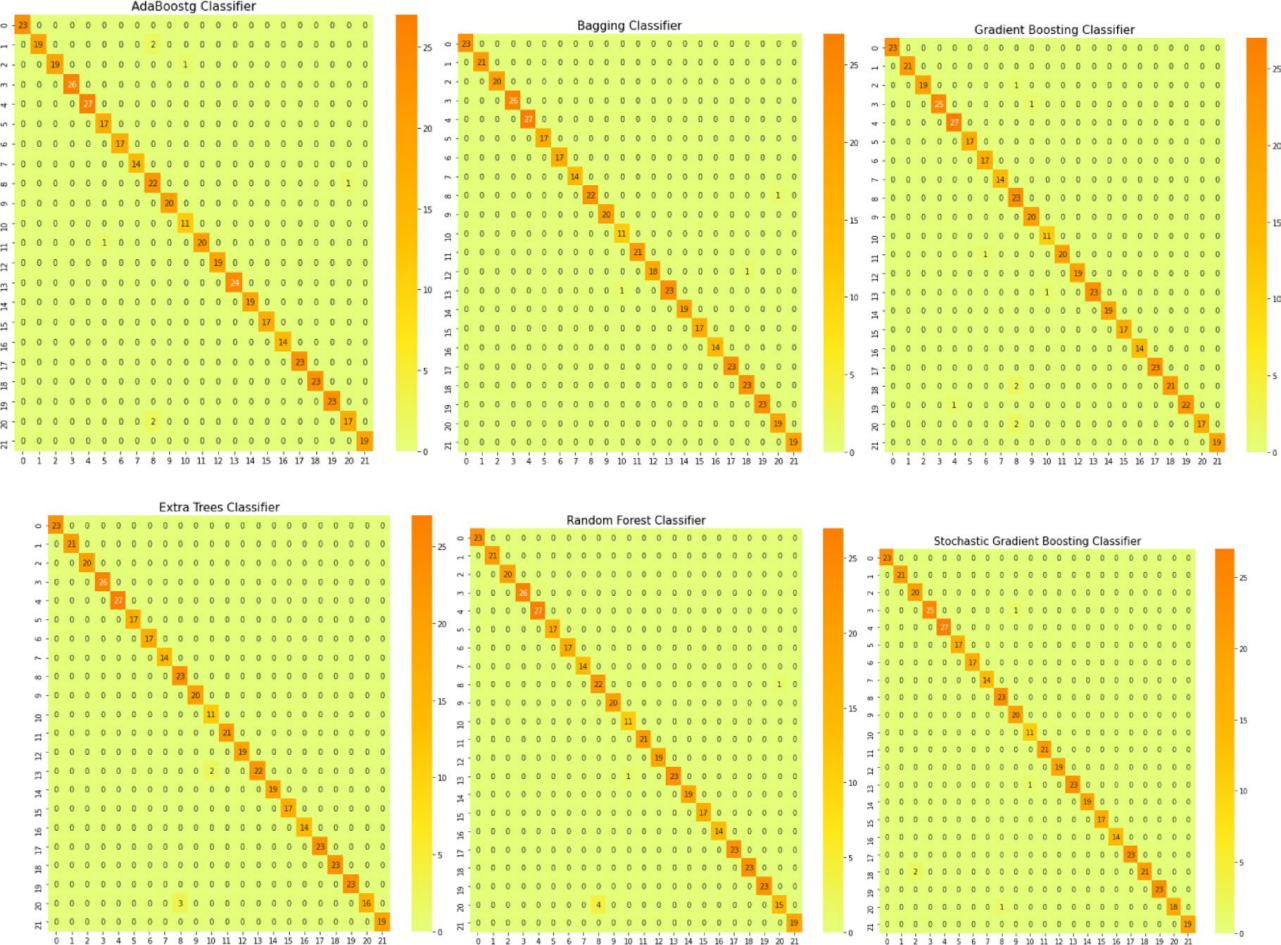
Confusion matrices for various ensemble models.

In this section, we provide a comprehensive analysis of three distinct ensemble models, each employing a different methodology. Figure 10 offers an insightful representation via a confusion matrix, which delineates the disparities between the proposed stacking models and the proposed max voting ensemble model. Intriguingly, three proposed ensemble models outshine the other six ensemble models, as vividly illustrated in Fig. 11.

**Fig. 12:**
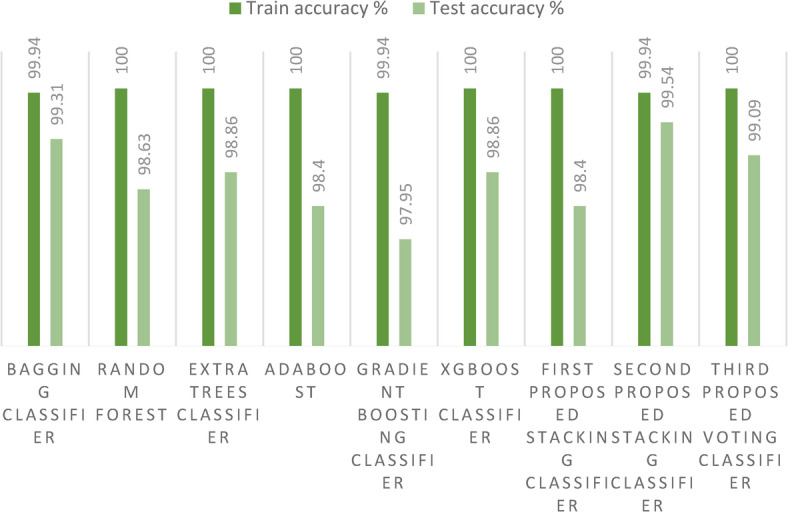
Training and test accuracies for the three proposed models and the ensemble models on the first dataset.

Turning our attention to Fig. 12, it graphically portrays the training and testing accuracies of the three avant-garde stacking models, juxtaposed with those of other ensemble models for the first dataset. A remarkable observation from the figure is that the training accuracies across all models peak at 100% or close to it. However, it is in the realm of test accuracy that we witness some differentiation. While the GB model and its associated methods trail with relatively lower accuracies due to overfitting, the stacking model emerges as the frontrunner.

Most notably, our second proposed stacking model achieves a stellar accuracy of 99.53%, greater than those of the other ensemble models. Furthermore, the Bagging model does not lag far behind, registering a commendable accuracy of 99.31%. The first proposed stacking model achieves the lowest accuracy of our proposed models. It was also observed that the overfitting in the three proposed models is reduced. Such enhancements undeniably bolster the predictive prowess of our crop recommendation system.

**Fig. 13:**
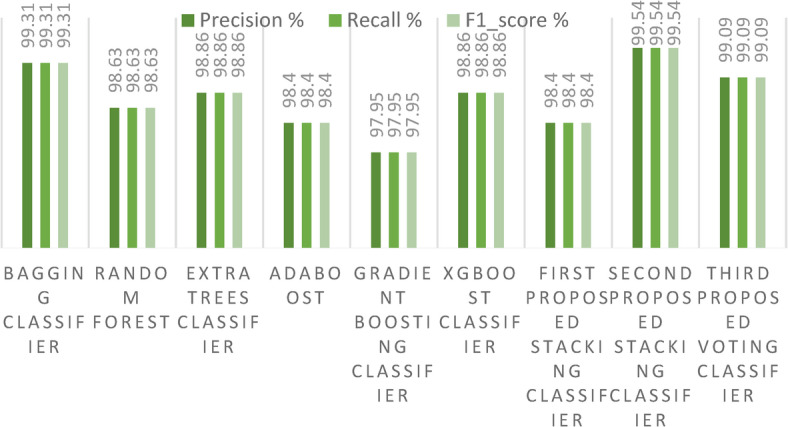
Precision, Recall, and F1-score for the three proposed models and the ensemble models for the first dataset.

In our first dataset analysis, Fig. 13 meticulously delineates the Precision, Recall, and F1-score values for the two avant-garde stacking models, benchmarking them against other ensemble models using the initial method of dataset division. One can discern from the figure that our second proposed stacking classifier excels over the other ensemble classifiers. Additionally, our proposed voting ensemble classifier demonstrates commendable results across Precision, Recall, and F1-score, showing particular prowess in scenarios that involve multi-class classification. A notable observation from the figure is the underperformance of the GB model in each metric, signaling its potential limitations in handling multi-class classification tasks. A comprehensive overview of performance metrics for these methods of dataset division, juxtaposing existing ensemble models with our innovative stacking ensemble models, is shown in Table 2.

**Table 2:** Comparison of performance metrics (%) between existing ensemble models and the proposed staking ensemble models on the first dataset.

Model	NA	TA	Pr	Re	F1-score	KC	HL	JS	MC
Bagging classifier	99.94	99.31	99.31	99.31	99.31	**99.28**	**0.68**	98.57	**99.28**
RF clasifier	**100**	98.63	98.63	98.63	98.63	98.56	1.36	97.45	98.57
ET classifier	**100**	98.86	98.86	98.86	98.86	98.80	1.13	97.67	98.81
Adaboost classifier	**100**	98.4	98.4	98.4	98.4	98.33	1.59	96.96	98.33
GB classifier	99.94	97.95	97.95	97.95	97.95	97.85	2.04	96.29	97.86
Xgboost classifier	**100**	98.86	98.86	98.86	98.86	98.80	1.136	97.80	98.81
First proposed stacking classifier	**100**	98.4	98.4	98.4	98.4	**99.28**	**0.68**	**98.59**	**99.28**
Proposed stacking classifier	99.94	**99.54**	**99.54**	**99.54**	**99.54**	98.33	1.59	96.99	98.33
Third proposed voting classifier	**100**	99.09	99.09	99.09	99.09	99.04	0.90	98.18	99.05

Significant values are in bold.

**Fig. 14:**
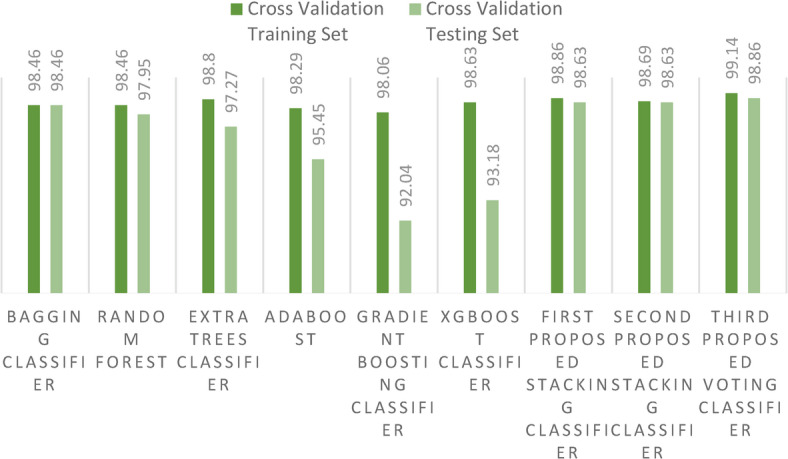
Accuracy score for the proposed models and the ensemble models on the first dataset using cross-validation strategy.

As we further dissect our findings, there emerges a pattern of occasional instability in outcomes, likely attributable to the inherent randomness when segmenting datasets into training and test sets. To bolster the reliability of our results, we embraced a secondary method of dataset division: cross-validation strategy using the *k*-fold approach with 10 folds. This stratagem yielded more consistent outcomes. Figure 14 sheds light on these results, highlighting the relatively modest performance of the GB classifier. In contrast, our proposed voting ensemble classifier stands out, achieving a laudable accuracy score of 98.86% with cross-validation strategy.

**Fig. 15:**
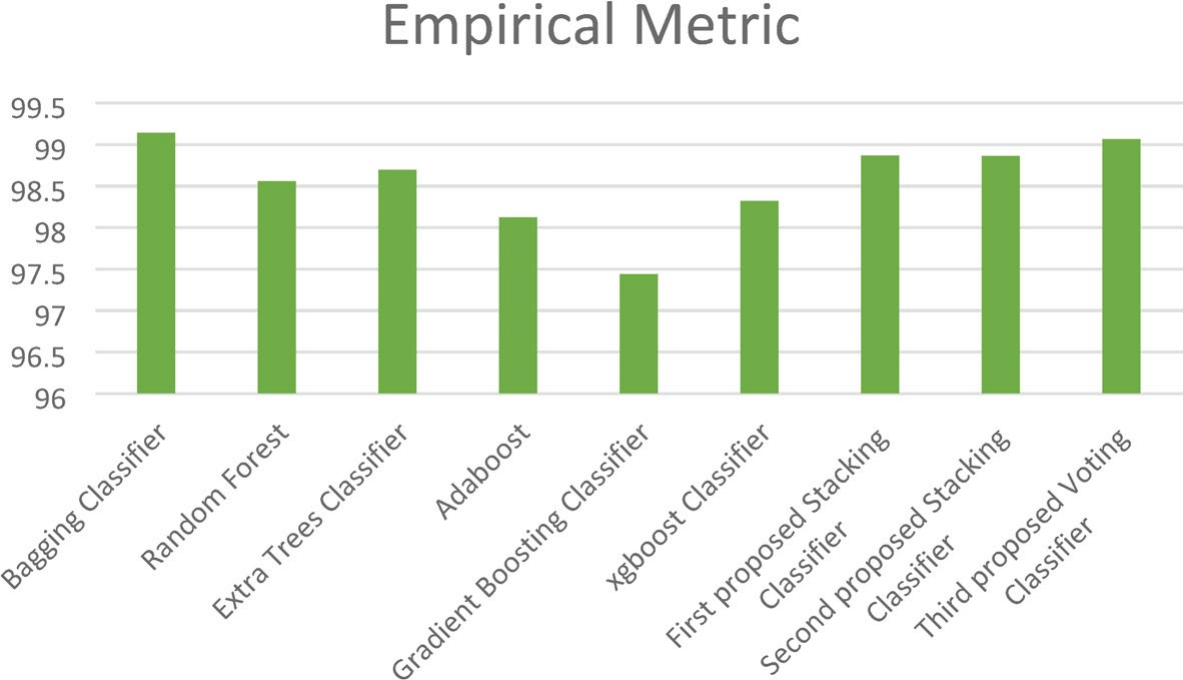
Empirical Metric (EM) for the three proposed models and the ensemble models on the first dataset.

Moreover, in this paper, we introduce a new metric (EM) that combines the complement of the Hamming loss and eight evaluation metrics (Training Accuracy, Testing Accuracy, Precision, Recall, F1_score, Cohen’s Kappa, Jaccard Score, and Matthews Correlation Coefficient) with equal weight to determine the best model as shown in Fig. 15. This figure shows the superiority of the three proposed stacking models to the ensemble models except the Bagging classifier, which has close results to those of the proposed models.

For a more granular understanding, Table 3 presents the outcomes using cross-validation strategy, juxtaposing the existing ensemble models with our proposed stacking ensemble model. Additionally, it shows the computational times of all models, revealing that the ET classifier has the least computational time, while the GB classifier has the highest computational time. This is due to complexity; however, the second proposed stacking model yields a less acceptable computational time of 11.43 s. The accuracy of detection for each crop with the three proposed staking ensemble models is also shown in Table 4.

**Table 3:** Comparison of existing ensemble models and the proposed stacking ensemble models on the first dataset using cross-validation strategy.

Performance metrics	Bagging classifier	RF classifier	ET classifier	Adaboost classifier	Gradient boosting classifier	Xgboost classifier	First proposed stacking classifier	Second proposed stacking classifier	Third proposed voting classifier
Cross-validation training set	98.46	98.46	98.80	98.29	98.06	98.63	98.86	98.69	**99.14**
Cross-validation test set	98.46	97.95	97.27	95.45	92.04	93.18	98.63	98.63	**98.86**
Empirical metric	**99.14**	98.55	98.69	98.12	97.43	98.32	98.86	98.86	**99.06**
Computational time (second)	5.53	3.37	**2.53**	2.83	111	69.3	13.3	11.43	29.3

Significant values are in bold.

**Table 4:** Model accuracy for the three proposed stacking ensemble models on the first dataset.

Crop	Model accuracy			Crop	Model accuracy		
	First proposed stacking classifier	Second proposed stacking classifier	Third proposed voting classifier		First proposed stacking classifier	Second proposed stacking classifier	Third proposed voting classifier
Apple	1.00	1.00	1.00	Lentil	0.92	0.92	0.92
Banana	1.00	1.00	1.00	Maize	1.00	1.00	1.00
Black gram	0.95	0.95	1.00	Mango	1.00	1.00	1.00
Chickpea	1.00	1.00	1.00	Moth beans	1.00	1.00	1.00
Coconut	1.00	1.00	1.00	Mung bean	1.00	1.00	1.00
Coffee	1.00	1.00	1.00	Muskmelon	1.00	1.00	1.00
Cotton	1.00	1.00	1.00	Orange	1.00	1.00	1.00
Grapes	1.00	1.00	1.00	Papaya	1.00	1.00	1.00
Jute	0.85	0.96	0.88	Pigeon peas	1.00	1.00	1.00
Kidney beans	1.00	1.00	1.00	Pomegranate	1.00	1.00	1.00
Watermelon	1.00	1.00	1.00	Rice	0.94	0.95	1.00

**Fig. 16:**
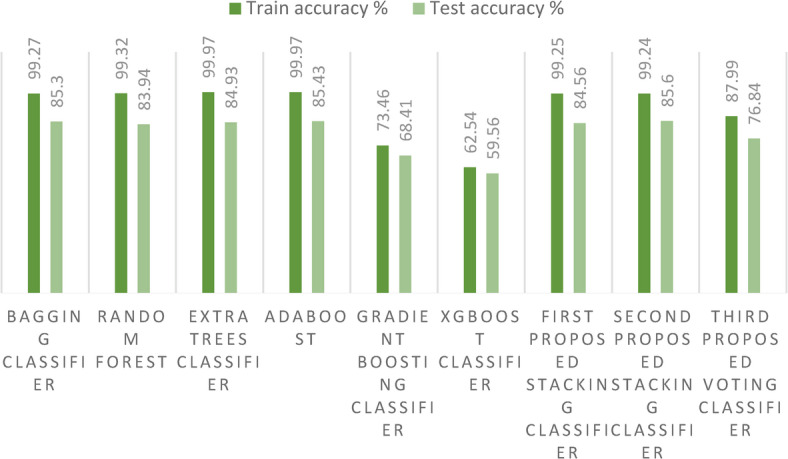
Training and test accuracies for the three proposed models and the ensemble models on the second dataset.

Turning our attention to Fig. 16, it graphically portrays the training and test accuracies of the three avant-garde stacking models, juxtaposed with those of other ensemble models on the second dataset. A remarkable observation from the figure is the differentiation between training and test accuracies. While the XGBoost classifier and its associated methods trail with relatively lower accuracies, mainly due to overfitting, the stacking model emerges as the frontrunner. Most notably, our second proposed stacking model, which incorporates the Bagging technique, achieves a stellar accuracy of 85.6%, surpassing the performance of other ensemble models. Furthermore, the Bagging model alone does not lag far behind, registering a commendable accuracy of 85.3%. Among the proposed models, the voting ensemble classifier records the lowest accuracy, indicating that it is not well-suited for large datasets.

**Fig. 17:**
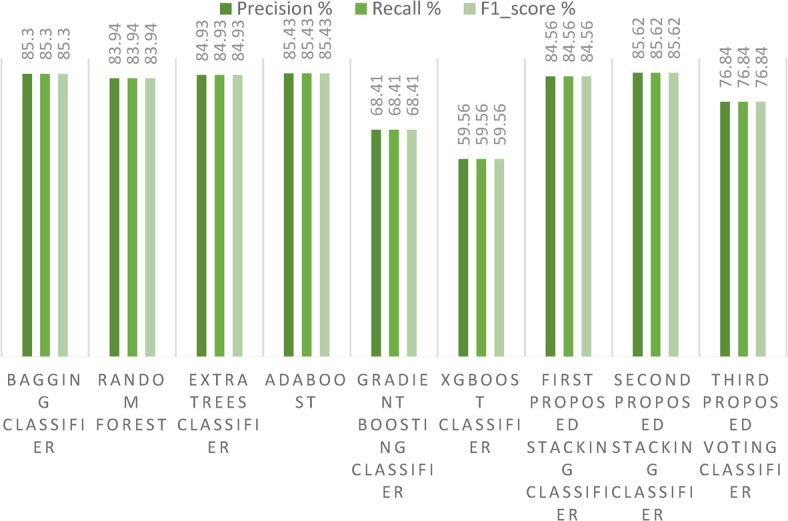
Precision, Recall, and F1-score for the three proposed models and the ensemble models on the second dataset.

In our second dataset analysis, Fig. 17 meticulously delineates the Precision, Recall, and F1-score metrics for the two avant-garde stacking models, benchmarking them against other ensemble models using the initial method of dataset division. As observed in the figure, our second proposed stacking classifier outperforms the other ensemble classifiers across all metrics. In contrast, the proposed voting ensemble classifier exhibits lower performance in terms of Precision, Recall, and F1-score, when applied to a large dataset. A notable observation is the consistent underperformance of the XGBoost classifier across all metrics, indicating its potential limitations in handling multi-class classification tasks on large datasets. A comprehensive overview of performance metrics for this dataset division method, juxtaposing existing ensemble models with our innovative stacking ensemble models, is provided in Table 5.

**Table 5:** Comparison of performance metrics (%) between existing ensemble models and the proposed staking ensemble models on the second dataset.

Model	NA	TA	Pr	Re	F1-score	KC	HL	JS	MC
Bagging classifier	99.27	85.30	85.30	85.30	85.30	83.32	14.69	72.23	83.32
RF classifier	99.32	83.94	83.94	83.94	83.94	81.77	16.05	70.34	81.7
ET classifier	**99.97**	84.93	84.93	84.93	84.93	82.89	15.06	72.30	82.9
Adaboost classifier	**99.97**	85.43	85.43	85.43	85.43	83.46	14.56	**73.20**	83.46
Gradient boosting classifier	73.46	68.41	68.41	68.41	68.41	64.09	31.58	51.3	64.16
Xgboost classifier	62.54	59.56	59.56	59.56	59.56	54	40.43	41.52	54.23
First proposed stacking classifier	99.25	84.56	84.56	84.56	84.56	82.47	15.43	71.51	82.48
Second proposed stacking classifier	99.24	**85.6**	**85.62**	**85.62**	**85.62**	**83.67**	**14.37**	72.82	**83.68**
Third proposed voting classifier	87.99	76.84	76.84	76.84	76.84	73.68	23.15	61.35	73.81

Significant values are in bold.

**Fig. 18:**
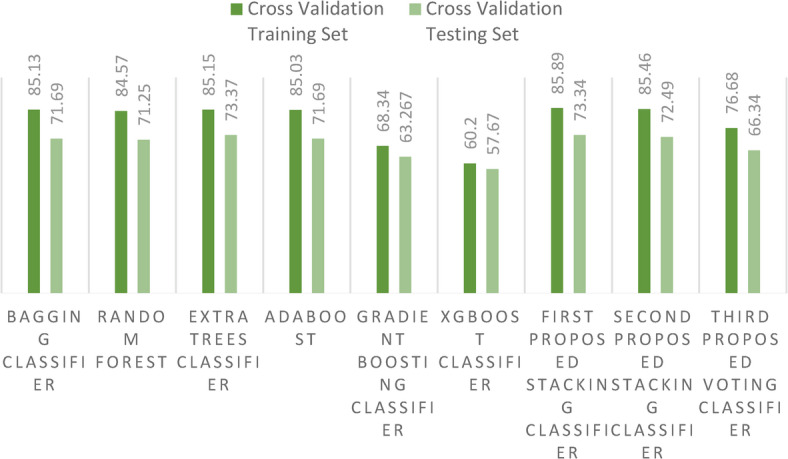
Accuracy scores for the proposed models and the ensemble models on the second dataset using cross-validation strategy.

**Fig. 19:**
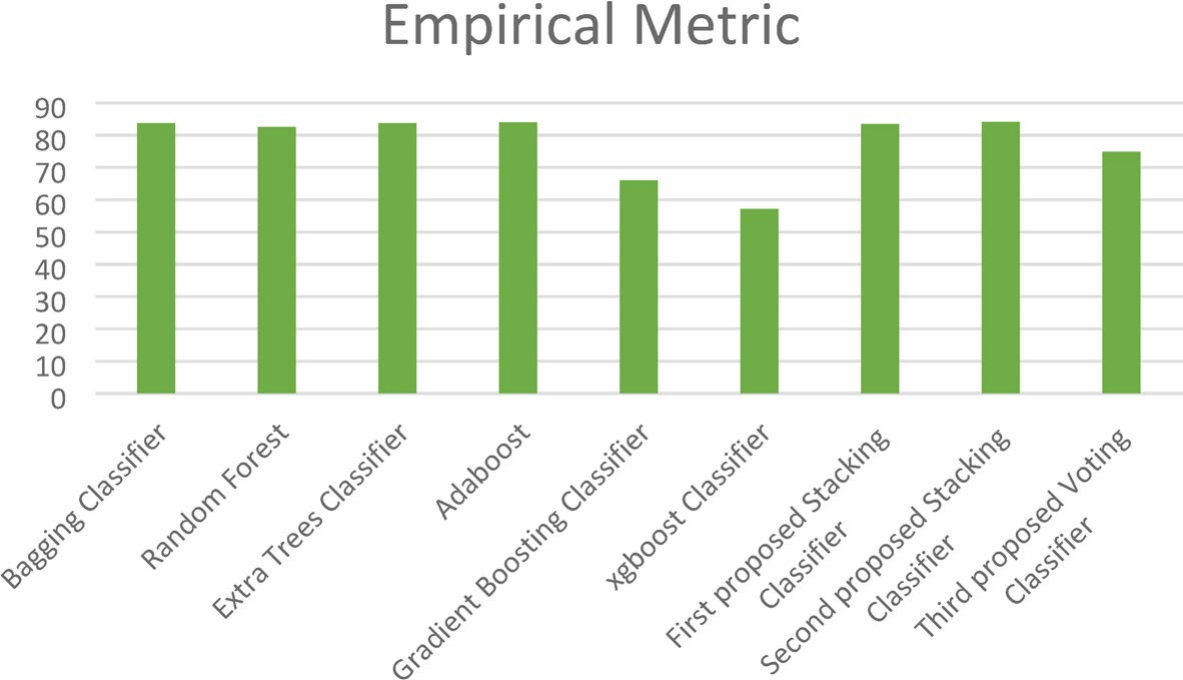
Empirical Metric (EM) values for the three proposed models and the ensemble models on the second dataset.

Figure 18 highlights the results of the cross-validation strategy using the *k*-fold approach with 10 folds, emphasizing the relatively modest performance of the GB classifier. In contrast, our first proposed stacking classifier stands out by achieving a laudable cross-validation score of 85.89%. Additionally, the new metric (EM) is illustrated in Fig. 19, further demonstrating the superiority of the two proposed stacking models over other ensemble models. For a more granular understanding, Table 6 presents the cross-validation outcomes, juxtaposing the existing ensemble models with our proposed stacking ensemble models. It also reports the computational times of all models, revealing that the ET classifier has the shortest computational time. Notably, the first proposed stacking model demonstrates an acceptable computational time of 41.4 s.

**Table 6:** Comparison of existing ensemble models and the proposed stacking ensemble model on the second dataset using cross-validation strategy.

Performance metrics	Bagging classifier	Random forest classifier	Extra trees classifier	Adaboost classifier	Gradient boosting classifier	Xgboost classifier	First proposed stacking classifier	Second proposed stacking classifier	Third proposed voting classifier
Accuracy with cross-validation training set	85.13	84.57	85.15	85.03	68.34	60.20	**85.89**	85.46	76.68
Accuracy with cross-validation test set	71.69	71.25	**73.37**	71.69	63.267	57.67	73.34	72.49	66.34
Empirical metric	83.77	82.60	83.74	83.99	66.06	57.08	83.43	**84.13**	74.91
Computational time (second)	30.4	7.31	**6.67**	331.5	829	334	41.45	235.8	273.5

Significant values are in bold.

## Comparison of ML models with the proposed models

**Fig. 20:**
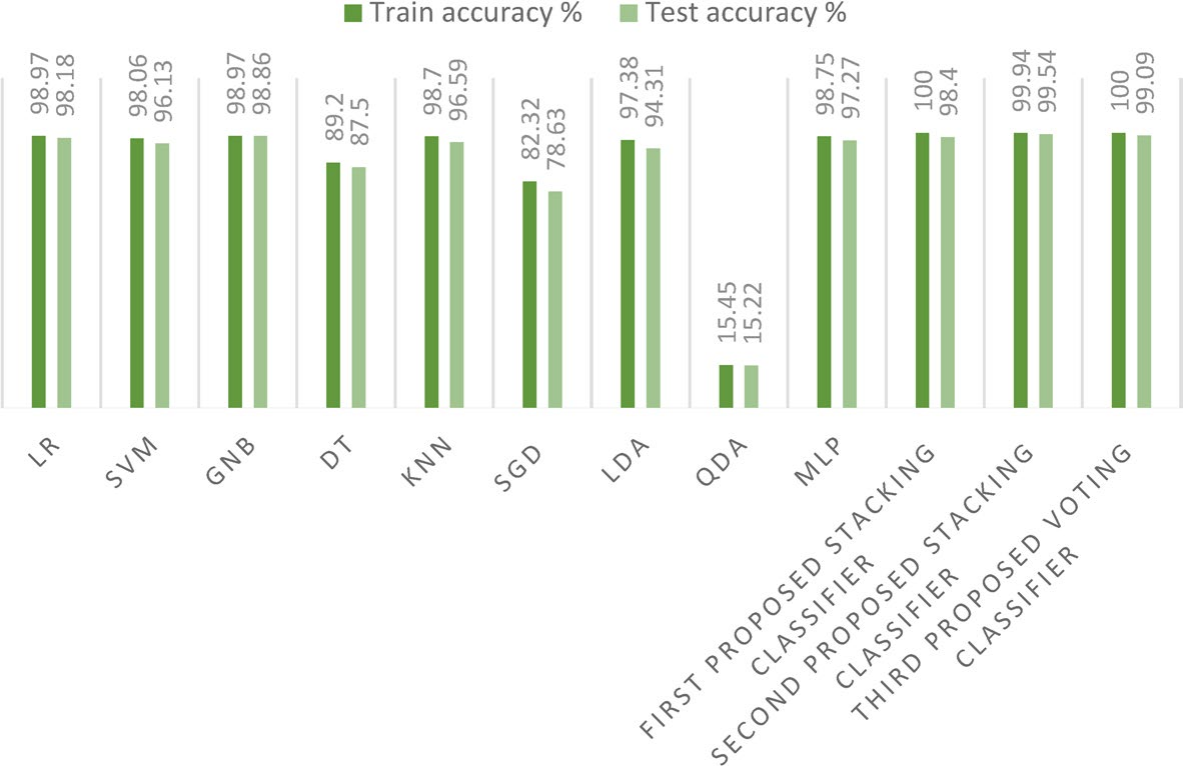
Comparison between the models’ training and test accuracies on the first dataset.

In this section, we conduct a comprehensive comparison between our three innovative ensemble models, specifically the stacking ensemble classifiers, and a suite of nine established ML models on the first dataset. As depicted in Fig. 20, a striking observation emerges: the training and test accuracies of the proposed stacking models are unparalleled, outperforming all nine ML models under consideration. This exceptional performance not only underscores the efficacy of our proposed models, but also significantly enhances the precision of the crop recommendation system, marking a notable advancement in this area of research.

**Fig. 21:**
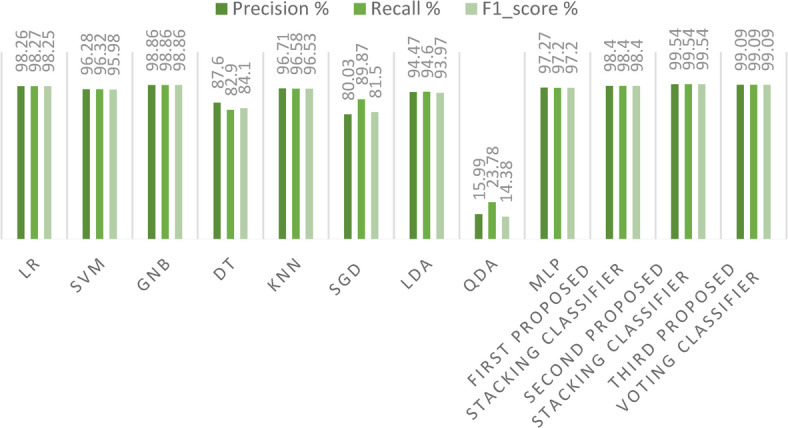
Precision, Recall, and F1-score for the three proposed models and ML models on the first dataset.

Figure 21 presents a detailed examination of Precision, Recall, and F1-score metrics for the avant-garde stacking models, juxtaposed against other ML models. It becomes clearly evident from the data that our proposed stacking classifiers excel in minimizing both false positive and false negative predictions. This exemplary performance yields F1-scores that are significantly superior to those of the other ML models. Table 7 meticulously catalogs all performance metrics related to our three cutting-edge stacking ensemble classifiers, comparing them with other ML models, particularly under the framework of the first primary dataset division method.

**Fig. 22:**
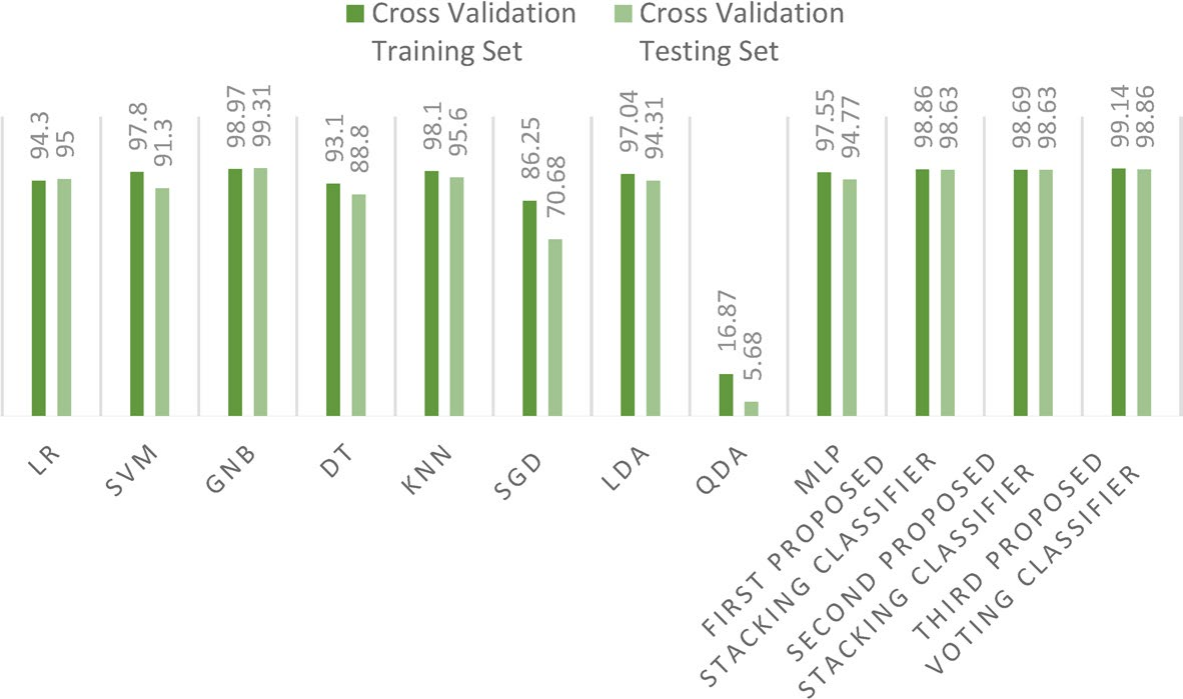
Comparison between accuracy scores for training and test using cross-validation strategy on the first dataset.

During our experimentation, we observed sporadic variations in outcomes when certain models were subjected to multiple iterations. This variability is attributable to the inherent randomness in the partitioning of training and test datasets. To address this issue and ensure more consistent results, we adopted a secondary dataset division strategy: cross validation with 10 folds. The outcomes derived from this robust cross-validation strategy for both training and test are illustrated in Fig. 22.

**Fig. 23:**
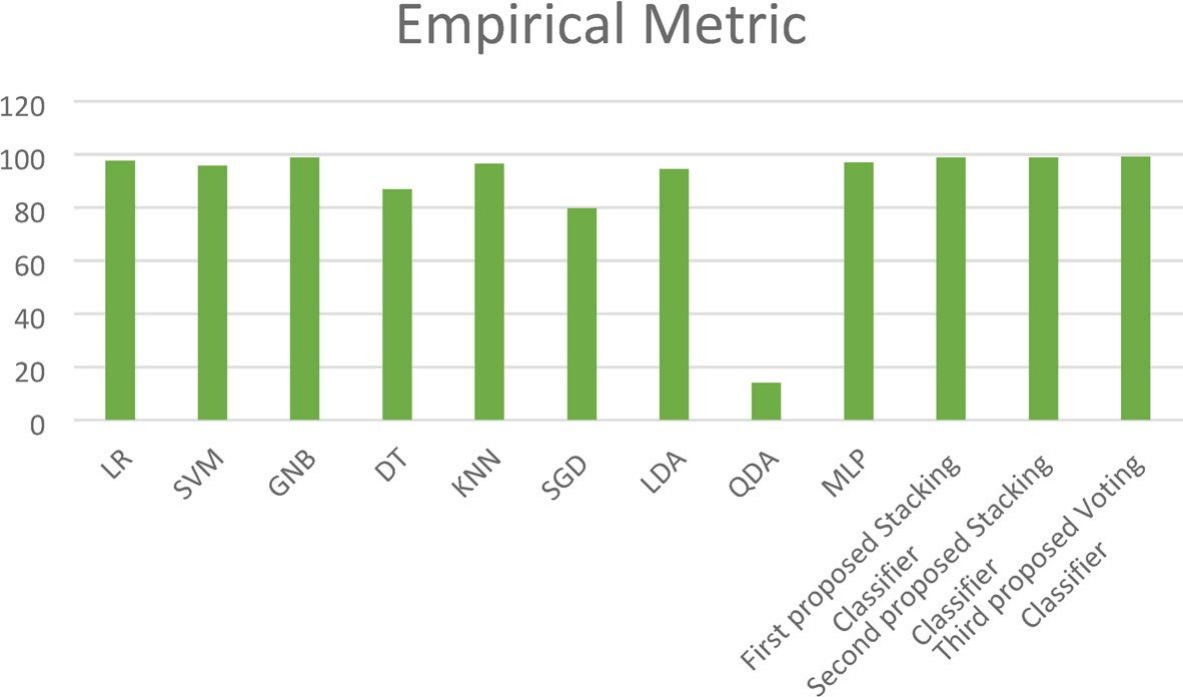
Empirical Metric (EM) values for the three proposed models and ML models on the first dataset.

Additionally, the paper introduces the EM, which is calculated to obtain average performance and identify the most effective model. As shown in Fig. 23, the EM demonstrates that the three proposed stacking models outperform the other ML models. For a comprehensive overview, Table 8 encapsulates the cross-validation and EM outcomes for our three proposed stacking ensemble classifiers in comparison with other established ML models. The table also reports the computational time of all models, showing that the LR classifier has the shortest runtime of 2.34 s, while the MLP classifier exhibits the highest. Notably, the second proposed stacking model achieves a reasonably acceptable computational time of 11.24 s, making it particularly suitable for offline training scenarios.

**Table 7:** Performance metrics (%) comparison of existing ML models and the proposed stacking ensemble models on the first dataset.

Model	NA	TA	Pr	Re	F1-score	KC	HL	JS	MC
LR classifier	98.97	98.18	98.26	98.27	98.25	98.09	1.81	96.82	98.09
SVM classifier	98.06	96.13	96.28	96.32	95.98	95.94	3.86	92.78	95.97
GNB classifier	98.97	98.86	98.86	98.86	98.86	98.80	1.13	97.78	98.81
DT classifier	89.2	87.5	87.6	82.9	84.1	86.88	12.5	80.17	87.27
KNN classifier	98.7	96.59	96.71	96.58	96.53	96.42	3.40	93.72	96.44
SGD classifier	82.32	78.63	80.03	89.87	81.50	77.58	21.36	72.40	78.41
LDA classifier	97.38	94.31	94.47	94.6	93.97	94.03	5.68	89.82	94.09
QDA classifier	15.45	15.22	15.99	23.78	14.38	11.58	84.77	8.10	11.89
MLP classifier	98.75	97.27	97.27	97.2	97.2	97.13	2.72	94.87	97.14
First proposed stacking classifier	**100**	98.4	98.4	98.4	98.4	**99.28**	**0.68**	**98.59**	**99.28**
Second proposed stacking classifier	99.94	**99.54**	**99.54**	**99.54**	**99.54**	98.33	1.59	96.99	98.33
Third proposed voting classifier	**100**	99.09	99.09	99.09	99.09	99.04	0.90	98.18	99.05

Significant values are in bold.

**Table 8:** Comparison of existing ML models and the proposed staking ensemble models on the first dataset using cross-validation strategy.

Performance metrics	LR	SVM	NB	DT	KNN	SGD	LDA	QDA	MLP	First proposed stacking classifier	Second proposed stacking classifier	Third proposed voting classifier
Cross-validation training set	94.3	97.8	98.97	93.1	98.1	86.25	97.04	16.87	97.55	98.86	98.69	**99.14**
Cross-validation test set	95	91.3	**99.31**	88.8	95.6	70.68	94.31	5.68	94.77	98.63	98.63	98.86
Empirical metric	97.49	95.7	98.81	86.82	96.54	79.66	94.39	22.07	96.94	98.86	98.86	**99.06**
Computational time (second)	**2.34**	3.8	3.9	4.1	2.5	4.48	4.58	4.6	38.6	13.3	11.43	29.3

Significant values are in bold.

**Fig. 24:**
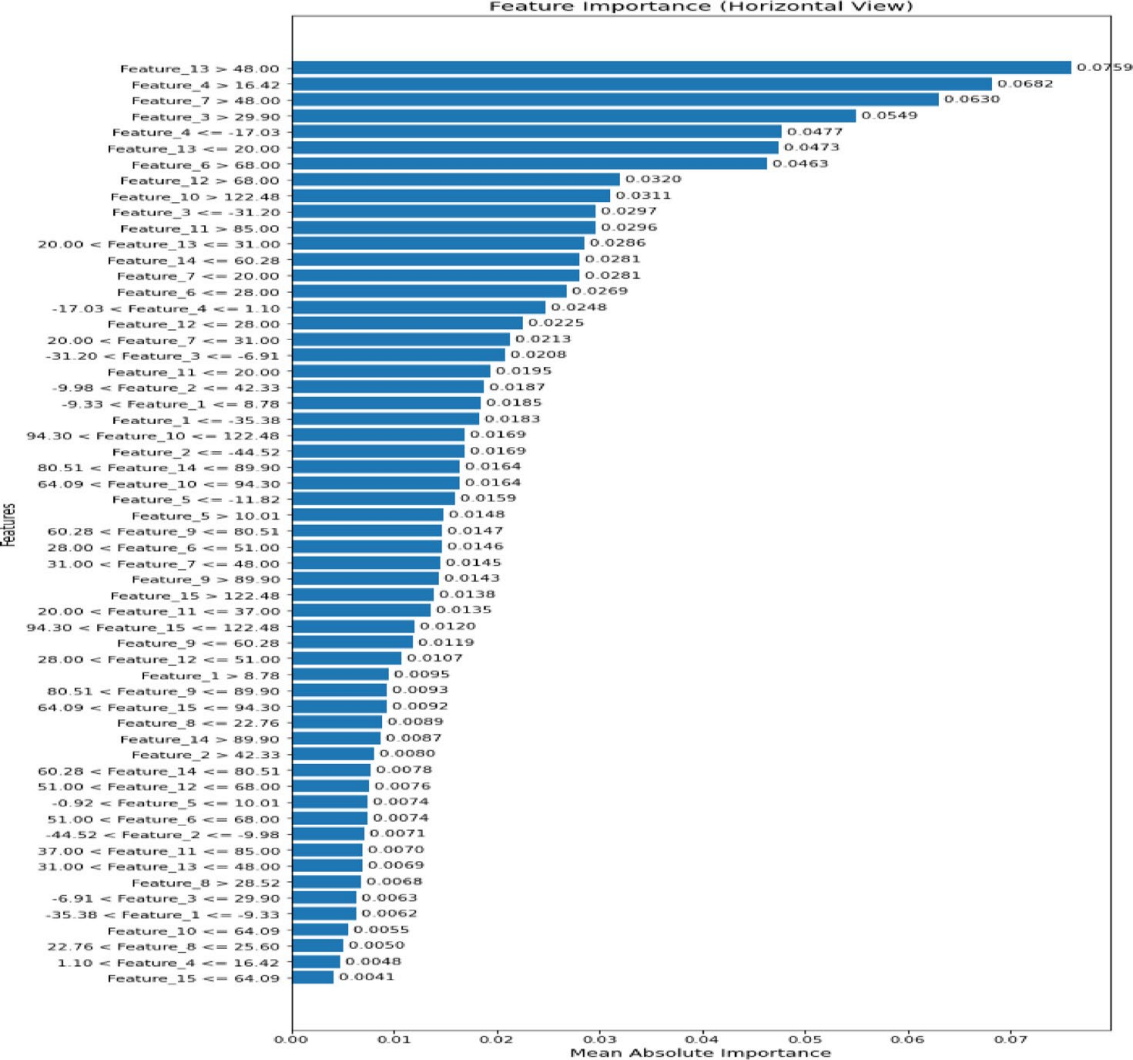
Importance of selected features for the third proposed voting classifier.

Figure 24, generated using Explainable Artificial Intelligence (XAI), illustrates the feature importance for the third proposed voting classifier, which achieved the highest accuracy on the first dataset. It illustrates the model crop recommendations by highlighting the most influential features based on mean absolute importance.

**Fig. 25:**
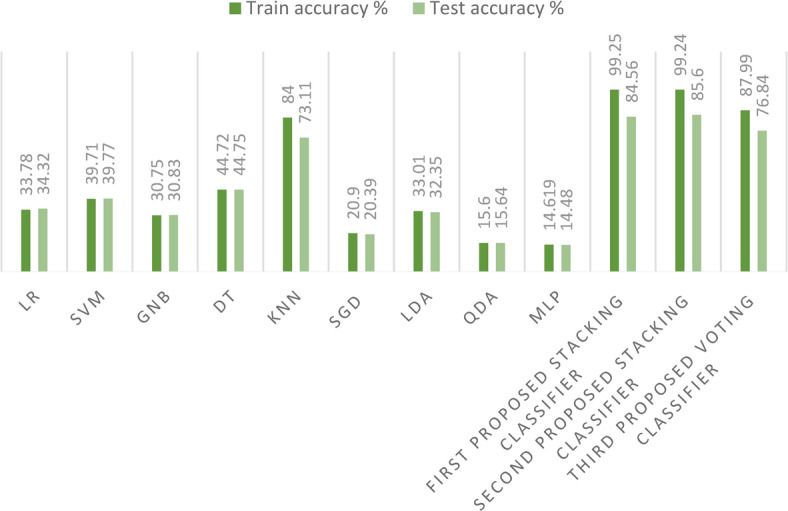
Comparison between the models’ training and test accuracies on the second dataset.

In this section, we also present a comparison between our three innovative stacking ensemble classifiers and a suite of nine established ML models on the second dataset. As in Fig. 25, a striking observation emerges: the training and test accuracies of the proposed stacking models outperform those of all nine other ML models, when applied on a large dataset. This outstanding performance not only underscores the efficacy of our proposed models, but also enhances the precision of the crop recommendation system, marking a significant advancement in this domain of research.

**Fig. 26:**
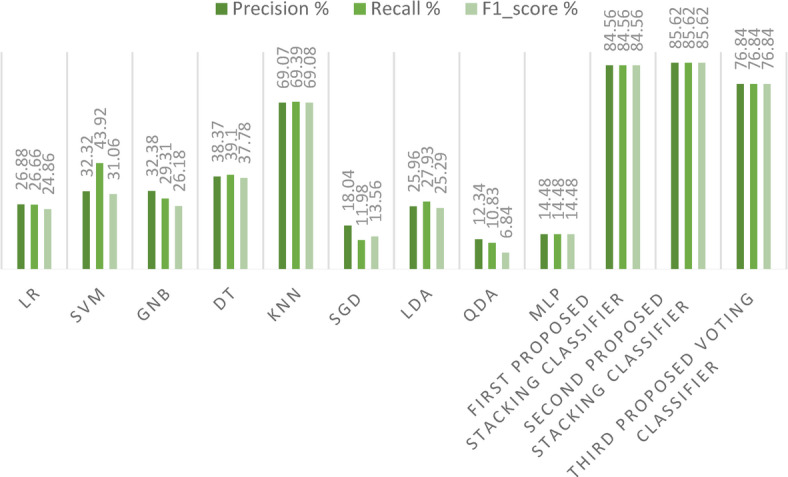
Precision, Recall, and F1-score for the three proposed models and the ML models on the second dataset.

Figure 26 presents a detailed examination of the Precision, Recall, and F1-score metrics for the avant-garde stacking models, juxtaposed to other ML models. It is clearly evident from the data that our proposed stacking classifiers outperform all other models, especially when applied to a large dataset. This indicates that the three proposed stacking classifiers perform acceptably and reliably at scale. This exemplary performance culminates in F1-scores that are markedly superior to those of the other ML models. Table 9 meticulously gives all performance metrics related to our three cutting-edge stacking ensemble classifiers, comparing them with other ML models.

**Table 9:** Comparison of existing ML models and the proposed stacking ensemble models on the second dataset.

Model	NA	TA	Pr	Re	f1	KC	HL	JS	MC
LR classifier	33.78	34.32	26.88	26.66	24.86	24.66	65.67	15.67	25.05
SVM classifier	39.71	39.77	32.32	43.92	31.06	30.97	60.223	19.80	31.41
GNB classifier	30.75	30.83	32.38	29.31	26.18	22.71	69.16	15.90	23.40
DT classifier	44.72	44.75	38.37	39.10	37.78	37.05	55.24	24.68	37.31
KNN classifier	84	73.11	69.07	69.39	69.08	69.45	26.88	53.78	69.48
SGD classifier	20.90	20.39	18.04	11.98	13.56	10.08	79.60	7.766	10.64
LDA classifier	33.01	32.35	25.96	27.93	25.29	22.36	67.64	15.76	22.76
QDA classifier	15.60	15.64	12.34	10.83	6.84	2.49	84.35	3.75	3.75
MLP classifier	14.619	14.48	14.48	14.48	14.48	0	85.51	1.44	0
First proposed stacking classifier	**99.25**	84.56	84.56	84.56	84.56	82.47	15.43	71.51	82.48
Second proposed stacking classifier	99.24	**85.6**	**85.62**	**85.62**	**85.62**	**83.67**	**14.37**	**72.82**	**83.68**
Third proposed voting classifier	87.99	76.84	76.84	76.84	76.84	73.68	23.15	61.35	73.81

Significant values are in bold.

**Fig. 27:**
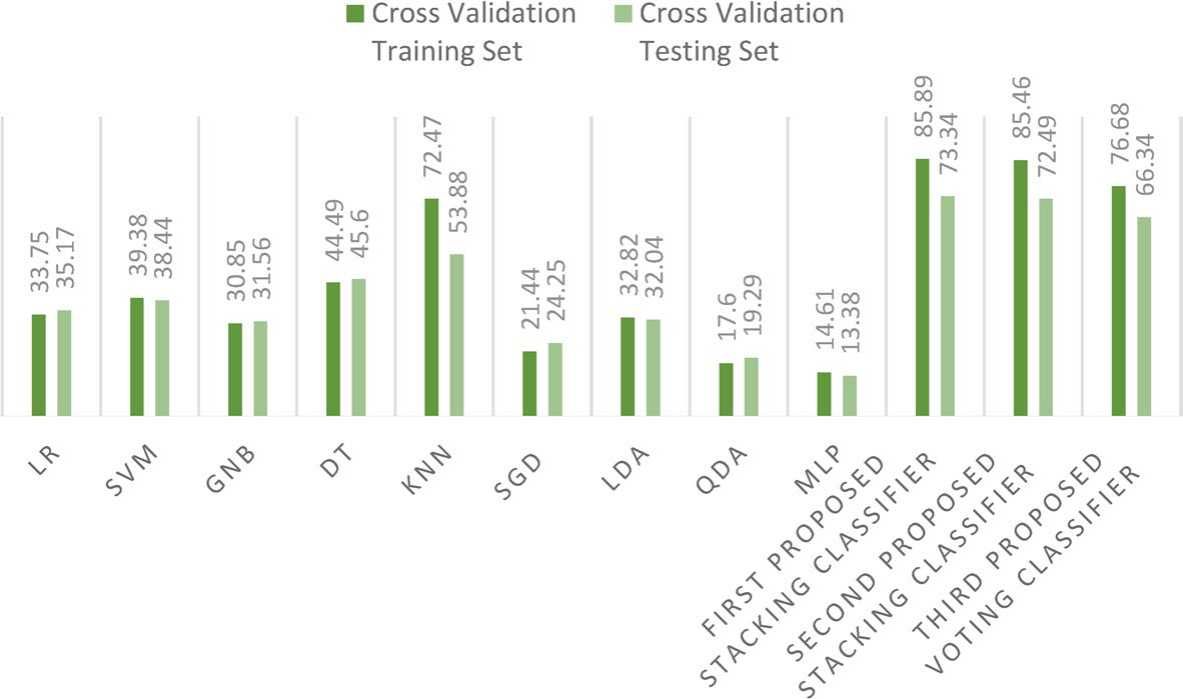
Comparison between existing ML models and the proposed stacking ensemble models on the second dataset using cross-validation strategy.

During our experimentation, we observed sporadic variations in outcomes when specific models were subjected to multiple iterations. To address this, we employed a secondary dataset division strategy: cross validation with 10 folds. The results derived from this robust cross-validation strategy for both training and test sets are vividly displayed in Fig. 27.

**Fig. 28:**
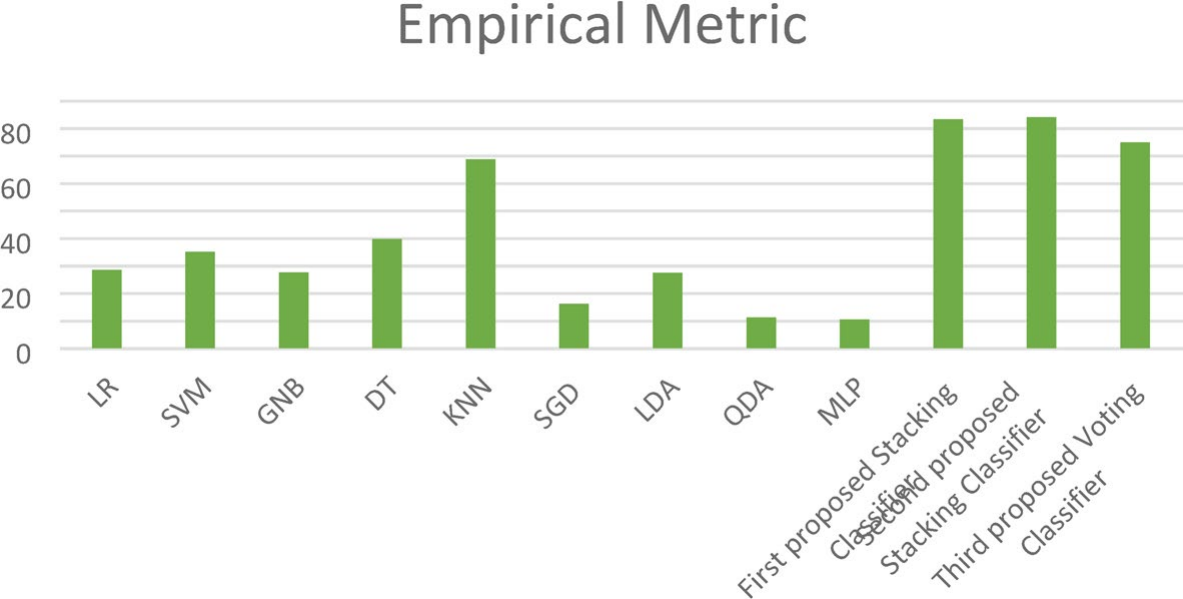
Empirical Metric (EM) values for the three proposed models and the ML models on the second dataset.

Additionally, the EM values, illustrated in Fig. 28, demonstrate that the three proposed stacking models outperform the other ML models. For a comprehensive overview, Table 10 encapsulates the cross-validation and EM results for our three proposed stacking ensemble classifiers in comparison with other established ML models. The table also includes the computational times for all models, showing that the LR classifier has the shortest runtime of 14.23 s, while the third proposed voting classifier records a longer computational time of 273.5 s.

**Table 10:** Comparison of existing ML models and the proposed stacking ensemble models on the second dataset using cross-validation.

Performance metrics	LR	SVM	NB	DT	KNN	SGD	LDA	QDA	MLP	First proposed stacking classifier	Second proposed stacking classifier	Third proposed voting classifier
Accuracy with cross-validation training set	33.75	39.38	30.85	44.49	72.47	21.44	32.82	17.60	14.61	**85.89**	85.46	76.68
Accuracy with cross-validation test set	35.17	38.44	31.56	45.60	53.88	24.25	32.04	19.29	13.38	**73.34**	72.49	66.34
Empirical metric	28.64	35.14	27.7	39.87	68.8	16.31	27.51	11.25	10.58	83.43	**84.13**	74.91
Computational time (second)	**14.23**	259	259	261	16.23	270	270	271	16.07	41.45	235.8	273.5

Significant values are in bold.

Finally, Table 11 presents a comparative analysis of our proposed models with contributions from previous studies, highlighting the advancements and innovations introduced in this research.

**Table 11:** Comparison between the proposed models and the previous models.

References	Contributions	Dataset	ML algorithms	Accuracy (%)
^26^	Utilization of sensors to collect temperature and humidity with ML models for a crop recommendation system and a mobile application for farmers	Crop dataset with features (N, P, K, T, humidity, rainfall)	KNN	97.7
			DT	99.7
			SVC	98.1
			NB	99
			RF	99.7
			GBM	98.8
			Extra tree	99.5
			XGB	99.3
^28^	Application of feature selection and feature extraction based on AI and a variety of ML algorithms for crop recommendation	Crop dataset with features (N, P, K, T, humidity, pH, rainfall)	LR	98.5
			DT	98.3
			GNB	96.9
			MNB	96.1
			GB	96.1
			SVM	98.5
			RF	99.1
			XGB	99
			Ridge regression	97.2
			Bagging	98.9
			SGD	98.5
			CBOOST	99.15
^35^	Examination of crop recommendation performance using seven different ML methods	Crop dataset with features (N, P, K, T, humidity, pH, rainfall)	LR	94.5
			DT	99
			RF	99.5
			KNN	98.6
			NB	99.5
			SVM	99.2
			Neural Network	97.7
^36^	Utilization of SMOTE to balance the dataset with ML models for a crop recommendation system	246,091 sample records with 37 different crops	LR	76
			DT	95
			KNN	94
			SVC	74
			RF	95
			Gradient Boost	94
			Bagged	96
			XGB	97
			AdaBoost	90
			Cat Boost	96
			HGB	96
			DGDC	100
			MNB	96
Our work	Application of three proposed stacking ensemble models with feature fusion for the crop recommendation system. Comparison of the proposed models with 9 ML models and 6 ensemble models. Providing 10 evaluation metrics and estimation of computational times to evaluate all models. Solving the overfitting problem, reducing computational time, and enhancing accuracy	Twenty-two crops with features (N, P, K, T, humidity, pH, rainfall)	First stacking ensemble model	**98.4**
			Second stacking ensemble model	**99.54**
			Third stacking ensemble model	**99.09**

Significant values are in bold.

**Fig. 29:**
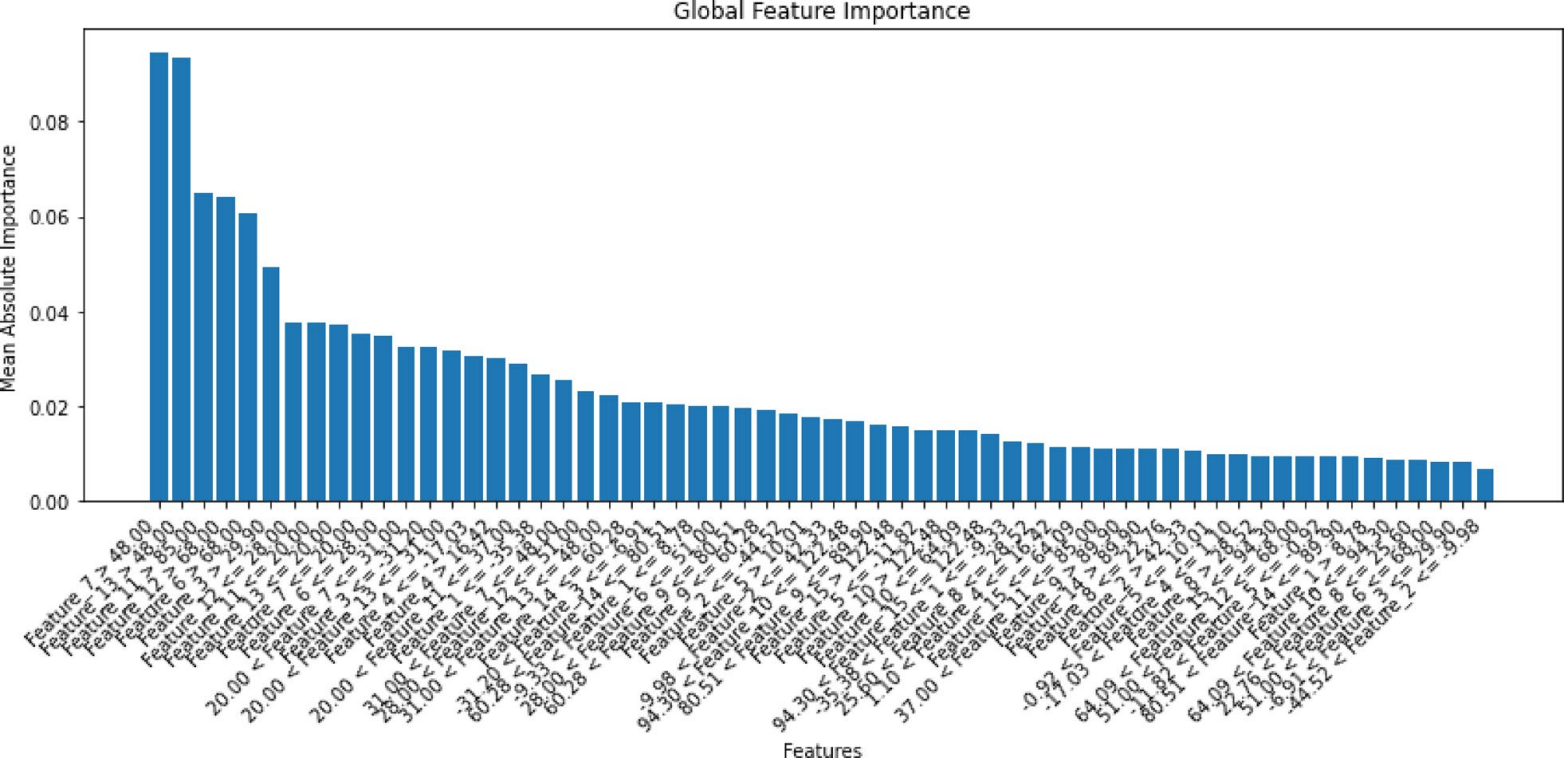
Importance of selected features by the second proposed stacking classifier.

Figure 29 was generated using XAI, illustrating the feature importance of the second proposed stacking classifier, which achieves the highest accuracy on the second dataset. It illustrates the model crop recommendations, highlighting the most influential features based on their mean absolute importance.

## Conclusions and future work

In this study, we introduced advanced stacking ensemble classifiers with feature fusion tailored for a crop recommendation system designed to guide farmers in making informed crop choices aligned with prevailing weather conditions and soil characteristics. Our research pivots on three innovative methods: two stacking ensemble classifiers and one voting ensemble classifier, all applied to two types of datasets for validation and verification. These methodologies not only systematically analyze the collected data, but also effectively identify the most optimal crop choices.

In terms of precision, the third proposed voting classifier emerged as a frontrunner, delivering outstanding accuracy compared to contemporary ensemble methods, particularly on the first dataset. Although this model helps reduce overfitting, it incurs the highest computational cost due to its complexity. For the second dataset, the second proposed stacking classifier achieved superior accuracy, especially in terms of the Empirical Metric (EM), when compared to other ensemble classifiers.

We also compared the performance of our models against Bagging, Boosting, and nine other established models: LR, NB, SVC, DT, KNN, LDA, QDA, SGD, and MLP. It is clear that each model integrated into our study demonstrated commendable effectiveness in recommending crops. However, a meticulous analysis of the results highlights the superiority of our proposed stacking models, which outperform the benchmarks set by previous ML models, elevating crop classification accuracy to an impressive value of 99.54%.

Looking ahead, a promising research direction lies in leveraging real-time datasets sourced directly from environmental sensors, enabled by the rapidly expanding Internet of Things (IoT) ecosystem, to generate predictive insights. Our future research aims to incorporate more comprehensive parameters—such as the specific water requirements for individual crops—which will play a crucial role in optimizing innovative irrigation systems. Moreover, in response to the evolving landscape of intelligent agriculture, we are developing a roadmap to integrate diverse datasets and combine our proposed models with deep learning architectures. This integration is expected to yield hybrid models that may redefine productivity benchmarks in precision farming.

## Data Availability

All data are available upon request from the corresponding author.
